# CO_2_-based production of phytase from highly stable expression plasmids in *Cupriavidus necator* H16

**DOI:** 10.1186/s12934-023-02280-2

**Published:** 2024-01-03

**Authors:** Simon Arhar, Thomas Rauter, Holly Stolterfoht-Stock, Vera Lambauer, Regina Kratzer, Margit Winkler, Marianna Karava, Robert Kourist, Anita Emmerstorfer-Augustin

**Affiliations:** 1 Austrian Centre of Industrial Biotechnology, acib GmbH, Krenngasse 37, Graz, 8010 Austria; 2 Institute of Biotechnology and Biochemical Engineering, Graz University of Technology, NAWI Graz, Petersgasse 12, Graz, 8010 Austria; 3 Institute of Molecular Biotechnology, Graz University of Technology, NAWI Graz, Petersgasse 14, Graz, 8010 Austria; 4 BioTechMed-Graz, Graz, Austria

**Keywords:** *Cupriavidus necator*, Segregational stability, Electroporation, Promoters, Phytase, Gas fermentation

## Abstract

**Background:**

Existing plasmid systems offer a fundamental foundation for gene expression in *Cupriavidus necator*; however, their applicability is constrained by the limitations of conjugation. Low segregational stabilities and plasmid copy numbers, particularly in the absence of selection pressure, pose challenges. Phytases, recognized for their widespread application as supplements in animal feed to enhance phosphate availability, present an intriguing prospect for heterologous production in *C. necator*. The establishment of stable, high-copy number plasmid that can be electroporated would support the utilization of *C. necator* for the production of single-cell protein from CO_2_.

**Results:**

In this study, we introduce a novel class of expression plasmids specifically designed for electroporation. These plasmids contain partitioning systems to boost segregation stability, eliminating the need for selection pressure. As a proof of concept, we successfully produced *Escherichia coli* derived AppA phytase in *C. necator* H16 PHB^− 4^ using these improved plasmids. Expression was directed by seven distinct promoters, encompassing the constitutive j5 promoter, hydrogenase promoters, and those governing the Calvin-Benson-Bassham cycle. The phytase activities observed in recombinant *C. necator* H16 strains ranged from 2 to 50 U/mg of total protein, contingent upon the choice of promoter and the mode of cell cultivation - heterotrophic or autotrophic. Further, an upscaling experiment conducted in a 1 l fed-batch gas fermentation system resulted in the attainment of the theoretical biomass. Phytase activity reached levels of up to 22 U/ml.

**Conclusion:**

The new expression system presented in this study offers a highly efficient platform for protein production and a wide array of synthetic biology applications. It incorporates robust promoters that exhibit either constitutive activity or can be selectively activated when cells transition from heterotrophic to autotrophic growth. This versatility makes it a powerful tool for tailored gene expression. Moreover, the potential to generate active phytases within *C. necator* H16 holds promising implications for the valorization of CO_2_ in the feed industry.

**Supplementary Information:**

The online version contains supplementary material available at 10.1186/s12934-023-02280-2.

## Background

*C. necator*, formerly known as *Ralstonia eutropha*, is a particularly attractive microbial host for the sustainable production of platform chemicals and proteins since it is able to capture CO_2_ via the Calvin-Benson-Bassham (CBB) cycle and convert it to biomass. (reviewed in [1, 2]) In order to stably introduce recombinant DNA into *C. necator*, conjugation and genomic integration have long been the method of choice, using *Escherichia coli* S17-1 as the donor bacterium. Conjugation, however, often results in low efficiencies, relatively long experimental times and does not allow for high-throughput screenings of genomic libraries. Therefore, new methods are currently on the rise that enable efficient electroporation and chemical transformation of plasmids [3–6]. Ideally, such plasmids shall be rather small, carry a broad host range (BHR) origin of replication, and a selectable marker. Following this principle, Azubuike et al. constructed the pCAT vectors, which can be either selected on kanamycin, tetracycline or chloramphenicol and, depending on whether or not they contained a recombinant gene, lead to transformation efficiencies of 10^3^ – 10^7^ cfu/µg DNA [7]. Additionally, it was reported that the deletion of the restriction endonuclease H16_A0006 could be a promising strategy to increase electroporation rates [8]. Episomal plasmids should also exhibit excellent segregational stability, and thereby be maintained within a population of cells over multiple generations. Segregational stability of plasmids largely depends on the used replication mechanism, copy number and selection pressure. The pBBR1 origin of replication is one of the replicons found most often on electroporation vectors. This replicon is rather small, leads to copy numbers between 7 and 40, and exhibits excellent segregational stability when selection pressure is applied. However, without selection pressure, 20% of *C. necator* cells lose the plasmid within four to eight 24 h serial passages of shake flask cultivations, depending on whether complex or minimal media are used [7, 9, 10]. The use of antibiotics to exert selective pressure on microbial cultures is subject to regulatory and ethical considerations. Overuse or misuse of antibiotics can contribute to the development of antibiotic-resistant strains of bacteria, which poses a significant global health threat. Also, antibiotics are expensive. Therefore, industry tries to avoid the use of antibiotics in fermentation processes. The classical approach for stable antibiotic free expression of target genes in *C. necator* is genomic integration, which is laborious to achieve due to low homologous recombination efficiencies and a conjugation-based methodology. An alternative strategy to reduce plasmid loss during antibiotic free cultivations is the usage of partitioning systems in episomal expression plasmids. This approach offers advantages due to faster delivery of target genes by electroporation and the potential for high copy number expression [11]. For example, the partitioning system from the RP4 plasmid increased stability for a pBBR1 based conjugational plasmid up to 95% over 4 days of antibiotic free cultivation [12]. The same study indicated an almost 100% stability for a vector utilizing the low copy number replicon from the pSa plasmid and the RP4 partitioning system. So far, electroporation vectors available for *C. necator* were only applied under antibiotic selection pressure, which is why this study aimed at establishing stable electroporation and expression vectors that can be used in antibiotic-free systems.

Besides its status as industrially relevant and well-studied production host for polyhydroxalkaloates, [2, 13] *C. necator* has been successfully engineered for the bioproduction of valued-added products, including for example alkanes [14], terpenoids [15, 16] and energy-containing molecules that might find application as biofuels (for a recent review see [17]). *C. necator* is also a powerful chassis for the production of industrial enzymes and proteins, due to its ability to suppress the formation of inclusion bodies, lack of acidic byproducts, and high fermentation cell density [18, 19]. Before the increasing popularity of soy products, *C. necator* was investigated as microbial protein source for its comparatively high protein content. However, due to concerns about accumulation of undigestible polyhydroxybutyrate (PHB) and elevated nucleic acid levels, which could potentially result in increased uric acid levels in consumers, *C. necator* was largely dismissed as a protein source for human and animal consumption. In recent years, the growing awareness of the climate crisis and the demand for eco-friendly high-quality protein production, coupled with *C. necator*’s capacity to thrive in mineral-based media using CO_2_ as a carbon source, have revived interest in its application as food and feed component [1, 20]. To valorize *C. necator* for the feed applications, we decided to produce the *E. coli* derived phytase AppA (AppA*Ec*) free of antibiotics, using novel, highly stable expression vectors in the PHB negative PHB^− 4^ strain. Phytases have the ability to break down phytate (IP6) into lower inositol phosphate forms (IP5-IP1) and inorganic phosphate. Thereby, phosphorous becomes accessible for monogastric animals. The degradation of phytate also increases concentrations of further essential minerals, since phytates as complexing agents reduce their biological availability. Phosphorus plays a crucial role in the diet of higher organisms because of its impact on bone formation and essential role in many cellular processes like energy metabolism and signal transduction [21, 22]. Monogastric animals (e.g. pigs and poultry), however, lack sufficient phytase activity in their digestive tract, which is why their diet usually requires supplementation with inorganic phosphate, although plant-based feed contains sufficient amounts of phosphorous in form of phytate. As a result, a large portion of this phosphate is excreted in their manure, which causes environmental pollution. Recent studies reported that feedstock supplementation with phytase (500 U/kg feed) can replace 0.1% NPP (non phytate phosphate) without affecting growth performance of broiler and Pekin ducks and reduce total amounts of phosphorus in the excreta by as much as 50% [23, 24]. Heterologous production of different phytases has successfully been achieved in *E. coli* and *P. pastoris* [25–27].

CO_2_ is an excellent feedstock for the generation of single cell protein for food and feed due to its affordability, lack of toxicity, and plentiful availability (with roughly 850 billion tons of CO_2_ already in the atmosphere). Also, it does not compete with the worldwide food production chain. In this study, we report the synthesis of the AppA*Ec* in *C. necator* using CO_2_ as sole carbon source. In our efforts to maximize AppA production using CO_2_, we strategically harnessed the transcriptional regulation intrinsic to *C. necator*’s chemolithoautotrophic lifestyle. Complementing this approach, we expedited plasmid delivery through electroporation, enabling the testing of multiple native promoters [28, 29]. The two best performing promoters were further employed in a lab-scale gas fermentation to confirm their performance and plasmid stability under industrial cultivation conditions.

## Results

### Construction of episomal expression vectors for protein production in *C. necator* H16

**Fig. 1:**
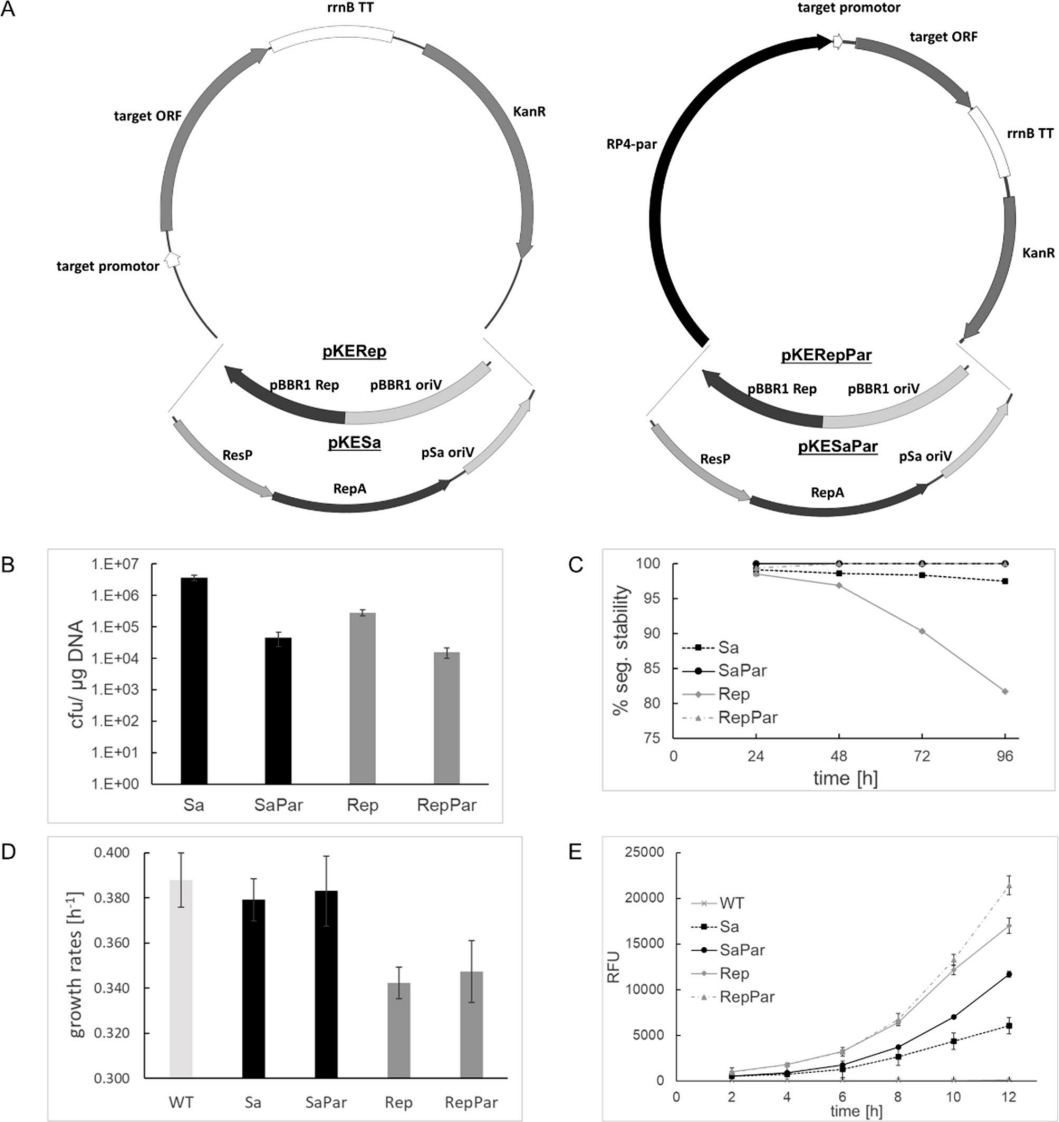
Construction and characterization of the electroporation vectors with and without the RP4 partitioning system. (**A**) Depictions of plasmids optimized for electroporation containing either the pBBR1 (pKERep) or the pSa (pKESa) replicon, which contain Rep and oriV or ResP, RepA and oriV respectively. To increase plasmid stability the RP4 partitioning system was added to create pKERepPar and pKESaPar. (**B**) Electroporation efficiencies for the four constructed plasmids (n = 3), obtained for *C. necator* H16 on TSB^Kan^ agar plates after 2 days at 28 °C. Presence of the plasmids was controlled by the applied selection and an eGFP derived green appearance of colonies. Error bars, SEM. (**C**) Segregational stability determination on antibiotic free TSB media at 28 °C. Cells were cultivated in 24 h intervals started by inoculation with the prior culture (n = 3). Loss of plasmid was determined by plating on TSB agar followed by a transfer of colonies to TSB^Kan^ plates and monitoring of the eGFP dependent green appearance of colonies. (**D**) Measurement of growth rates and (**E**) eGFP derived fluorescence during a 12 h cultivation of *C. necator* H16 carrying the four constructed plasmids (n = 3). Wild type *C. necator* H16 carrying no plasmid (WT) was used as a control. Cultures grew on antibiotic free TSB media at 28 °C as described in Materials and Methods. Error bars, SEM

In order to improve the segregational stability of expression plasmids, we combined the RP4 partitioning system with the pBBR1 (Rep, oriV) or the pSa (ResP, RepA, oriV) replicon on plasmids constructed for electroporation (Fig. 1, A). Plasmids were based on previously reported vector systems from Gruber et al. [12]. The main modification was the removal of all conjugation related parts to decrease plasmid size. The four new plasmids were named pKESa (4.7 kb), pKESaPar (6.9 kb), pKERep (3.7 kb) and pKERepPar (5.9 kb) (plasmid sequences available in Additional file 1). All four plasmids contained eGFP as model ORF and a APH(3’) -1 family aminoglycoside O-transferase mediating a resistance to kanamycin. The pKESa and pKESaPar plasmids contained the pSa replicon and the pKERep as well as pKERepPar the pBBR1 replicon, respectively. The RP4 partitioning (Par) system was additionally introduced to the pKESaPar and the pKERepPar plasmid.

The resulting plasmids were isolated from *E. coli* Top10 and *C. necator* H16 wildtype strain was transformed with them for further characterization. Electroporation rates for the new plasmids varied from ~ 4 × 10^6^ cfu/µg found for the pKESa plasmid to 1.6 × 10^4^ cfu/µg for pKERepPar (Fig. 1, B). Presence of the partitioning system decreased transformation rates for the pBBR1 and the pSa vector replicons (p < 0.0001). In general, the pSa based vectors yielded higher electroporation rates, which was surprising, since they were approximately 1 kb larger than the corresponding Rep plasmids (p ≤ 0.01). Segregational stability of the plasmids was determined during four serial passages on tryptic soy broth lacking any antibiotic for selection (Fig. 1, C). After the fourth passage, ~ 2% of the *C. necator* cells lost the pKESa vector. For the pKERep vector, an increasing percentage of cells lost the plasmid with every passage leading to a final loss of 20%. In contrast, 100% stability was observed for plasmids containing the RP4 partitioning system.

To further evaluate the constructed plasmids for their potential as expression vectors, twelve-hour cultivations on liquid tryptic soy media were performed (for the growth curves see Additional file 1. Figure S1). Compared to the wild type *C. necator* H16 strain, only slightly decreased growth rates from 0.39 ± 0.01 h^− 1^ for the wild type to 0.34 ± 0.01 h^− 1^ were found with the high copy number Rep plasmids (Fig. 1, D). Expression of eGFP with the constitutive t5 promoter was monitored via fluorescence measurements on a plate reader (Fig. 1, E). For all four plasmids, an increase in fluorescence was detected during the whole 12 h of cultivation. In general, the two Rep plasmids constantly showed 2–3 times higher eGFP levels than the Sa plasmids (p < 0.001), which may result from higher copy numbers.

### Evaluation of promoters for phytase expression in *C. necator* H16 PHB^-4^

**Fig. 2:**
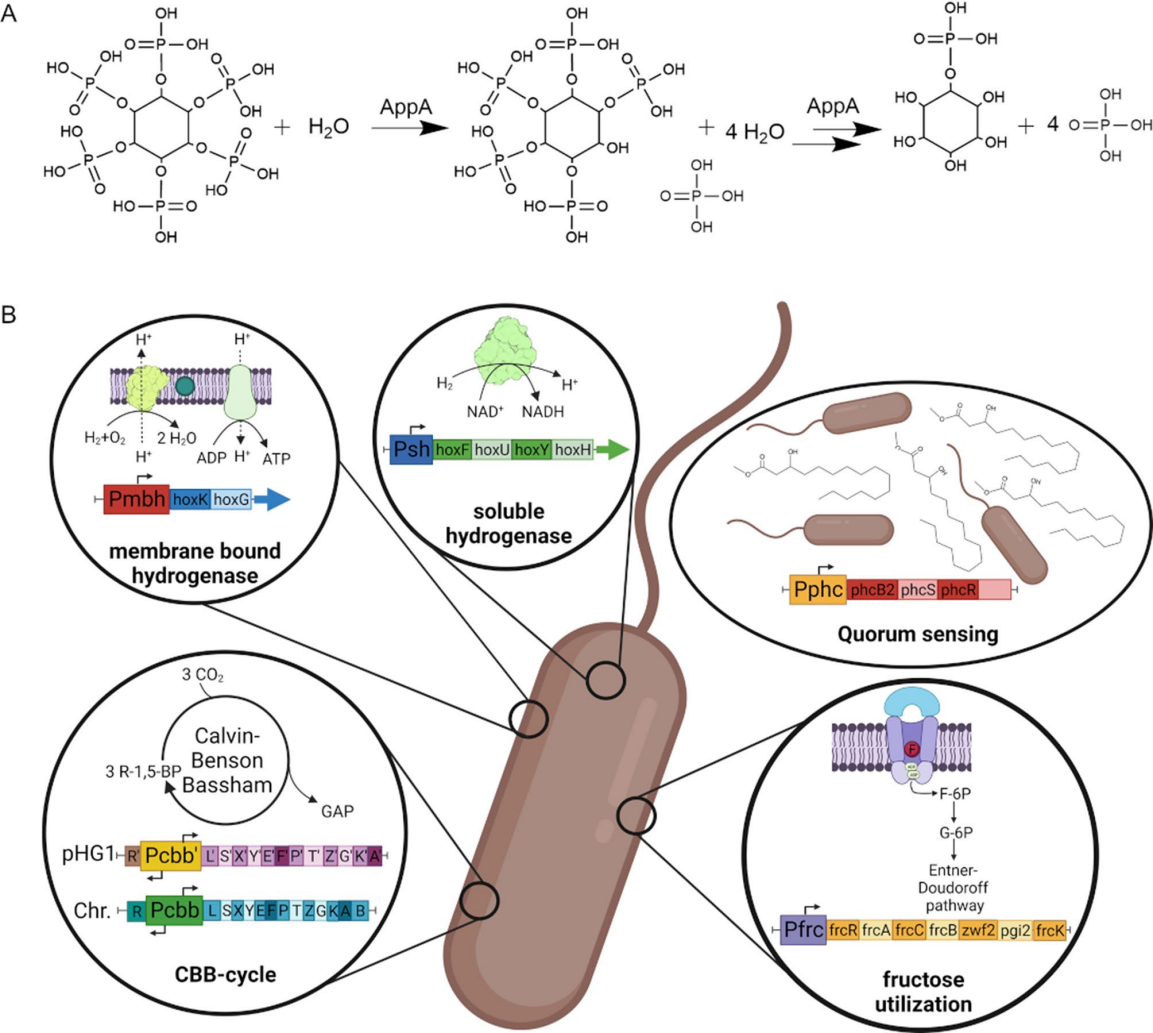
*E. coli appA* is expressed from different promoters and plasmids in *C. necator* H16 PHB^-4^. (**A**) AppA possesses classical phytase activity leading to the removal of one phosphate from plant derived phytic acid, and a phosphatase activity responsible for the release of up to four additional phosphate molecules. (**B**) Overview of the six native *C. necator* promoters tested for phytase production. Among them are the promoters of the two megaplasmid- and chromosomal-derived CBB-operons (cbb_pHG1 and cbb_chr), the promoters for the membrane bound and the soluble hydrogenase (mbh and sh), the promoter of a putative quorum sensing operon (phc) and the promoter of a fructose utilization operon (frc) (clockwise starting from the left, bottom position)

AppA*Ec* exhibits phytase activity and phosphatase activity, leading to consecutive removal of phosphates from a phytic acid molecule (Fig. 2, A). To avoid the accumulation of PHB, which is undigestible for most mammals, the PHB deficient *C. necator* PHB^− 4^ (DSM 541) strain was used for production of AppA*Ec*. Due to the high stability of the pKERepPar plasmid, we decided to use it as backbone for recombinant gene expression. Therefore, the *eGFP*-ORF was replaced by wild type *appA* from *E. coli* MG1655 and put under the control of different promoters (Fig. 2, B). The constitutive P_j5_ is commonly used and considered as strong promotor in *C. necator* [12, 30, 31] and was therefore used as a benchmark for AppA*Ec* expression. Due to their activation under lithoautotrophic conditions, we tested the well described promoters of the two CBB operons found on chromosome 2 (cbb_chr.) and the megaplasmid (cbb_pHG1). Furthermore, the promoter of the soluble hydrogenase found upstream of *hoxF* (sh) and the promoter of the membrane bound hydrogenase found upstream of *hoxK* (mbh) was evaluated to drive gene expression [28, 29, 32]. We further included a promoter found upstream of the putative Phc-quorum sensing operon, which might lead to cell density dependent *appA* expression (phc) [33]. The last promoter we tested was derived from the sequence upstream of the operon coding for the fructose transporter and the initial steps of fructose degradation (frc) [34]. Promotor sequences introduced into the new vectors were chosen by considering previously published studies characterizing the organization of corresponding operons and general structure of the annotated ORFs in the vicinity of target promotors. The presence of motives fitting to bacterial promotors was additionally verified by a promotor prediction tool based on neural networks [35].

**Fig. 3:**
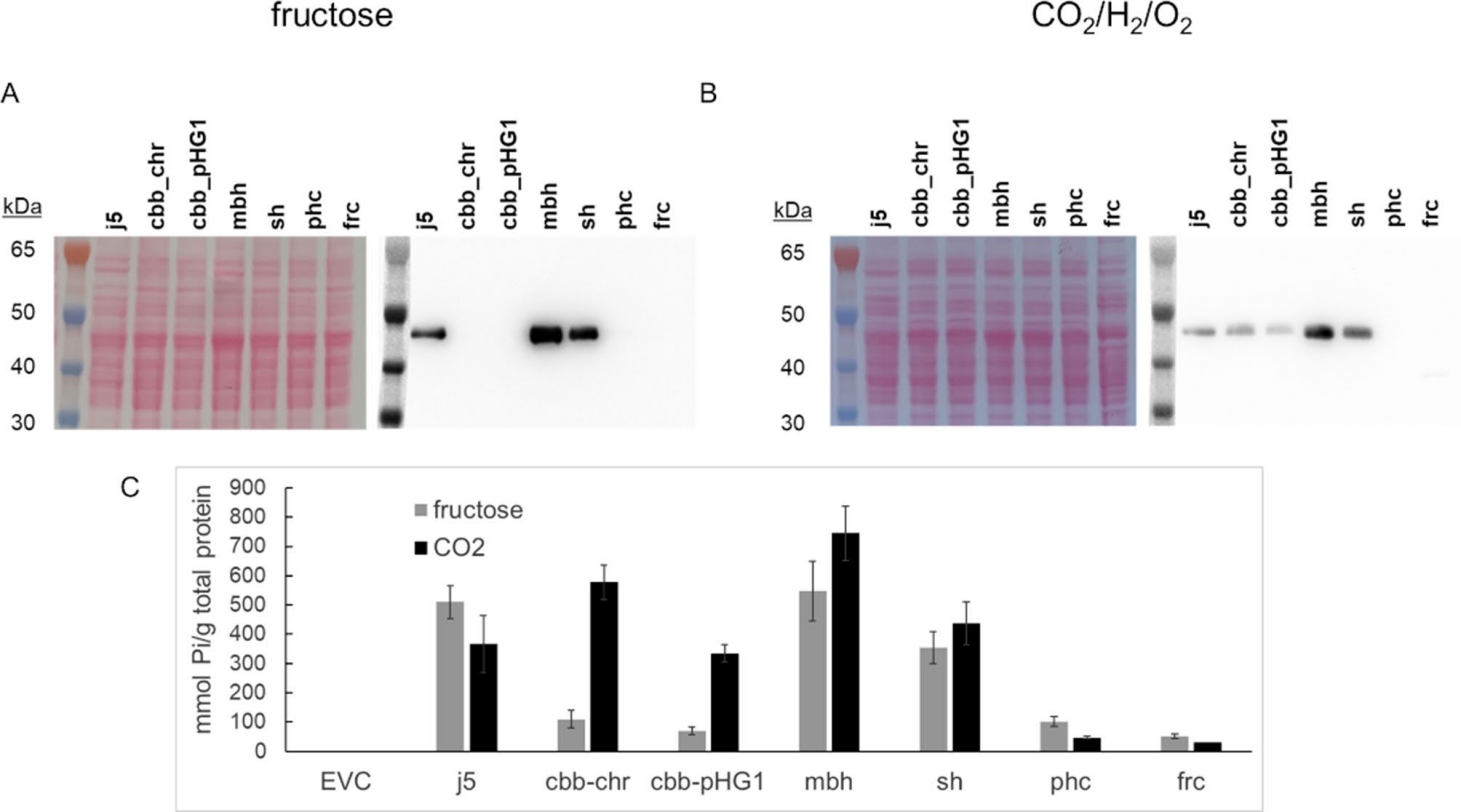
Promoter screening for heterotrophic and chemolithoautotrophic *E. coli* AppA production in *C. necator* H16 PHB^-4^. Cells were cultivated in mineral media containing kanamycin at 28 °C in baffled shake flasks till stationary phase. For heterotrophic growth, fructose was used as a carbon source. Chemolithoautotrophic cultivations were performed in an anaerobia pot under CO_2_/H_2_/O_2_ atmosphere. AppA expression levels with the different promoters were monitored by immunoblot with fructose (**A**) and CO_2_ (**B**) as carbon source. As a reference, the strong constitutive j5 promoter was used together with the *C. necator* derived promoters. Native promoters of the Calvin-Benson-Bassham operons (cbb_chr; cbb_pHG1), the membrane bound (mbh) and the soluble (sh) hydrogenases, a fructose degradation (frc) and a putative quorum sensing (phc) operon. As loading control, a Ponceau S staining of the membrane is shown. Phytase/phosphatase activity in the cell lysates (**C**) was determined by reactions with phytic acid as substrate (15 min, 37 °C) and a photometric measurement of the released phosphate. No background activity was found with an empty vector control (EVC)

Plasmids with the seven promoters were cloned and introduced into *C. necator* H16 PHB^− 4^ by electroporation. The corresponding clones were cultivated on mineral media containing fructose or a CO_2_/H_2_/O_2_ atmosphere as substrates for growth under heterotrophic and autotrophic conditions, respectively. Kanamycin was added to ensure plasmid stability during the promoter testing. With fructose as carbon and energy source, only j5, mbh and sh promoters led to detectable production of AppA according to immunoblot analysis (Fig. 3, A). This was surprising, since the two hydrogenase promoters were expected to be, at least, partially repressed on fructose [12, 29]. When examining the same strains for phytase activities, we noticed a comparable pattern as seen in the immunoblot analysis (Fig. 3, C). On fructose, highest AppA activities were detected when gene expression was driven by the mbh and j5 promoters, which both led to the release of ~ 500 mmol phosphate/g total protein. With CO_2_/H_2_/O_2_ as carbon and energy source, expression could also be achieved from the chromosomal and the pHG1 derived CBB promoters (Fig. 3, B**)**, which resulted in 3–5 times higher phytase activities (p = 0.0002) in the respective cell lysates (Fig. 3, C). Highest expression was again found for the weakly induced mbh promoter, leading to a phosphate release of 745 mmol/g total protein. Autotrophic growth marginally reduced activation of the constitutive j5 promoter, which only yielded 50% of the activity found with the mbh promoter (p = 0.008). In general, similar activities were observed for the sh and megaplasmid derived CBB promoter. The AppA activities obtained when gene expression was driven from the phc and frc promotors were low and phc showed a further decrease under lithotrophic growth (p = 0.01). Under heterotrophic and chemolithotrophic conditions, a similar final biomass was accumulated for most strains (Additional file Figure S2). A possible growth defect was mainly indicated with the j5 promotor, which accumulated only half of the biomass than obtained for the EVC or when phytase was produced from other promoters.

### Gas fermentation of *C. necator* H16 PHB^-4^ to produce AppA

For upscaling and to test vector stability under controlled cultivation conditions, we cultivated the most promising AppA production strains utilizing the mbh and the cbb_chr. promotors in a lab scale gas fermentation system [36, 37]. Cultivations were performed with mineral media containing 5 g/l of ammonium sulfate and a marginally lower buffer concentration as for the shake flask cultivations to decrease osmotic stress on *C. necator* H16 PHB^− 4^. The carbon dioxide feed and the total gas flow were kept constant, while O_2_ and H_2_ partial pressure was varied to maintain 2% dissolved oxygen, which was recently reported as optimal for biomass formation [37]. Ammonia concentration, OD_600_ and pH were monitored as additional process parameters by daily sampling.

**Fig. 4:**
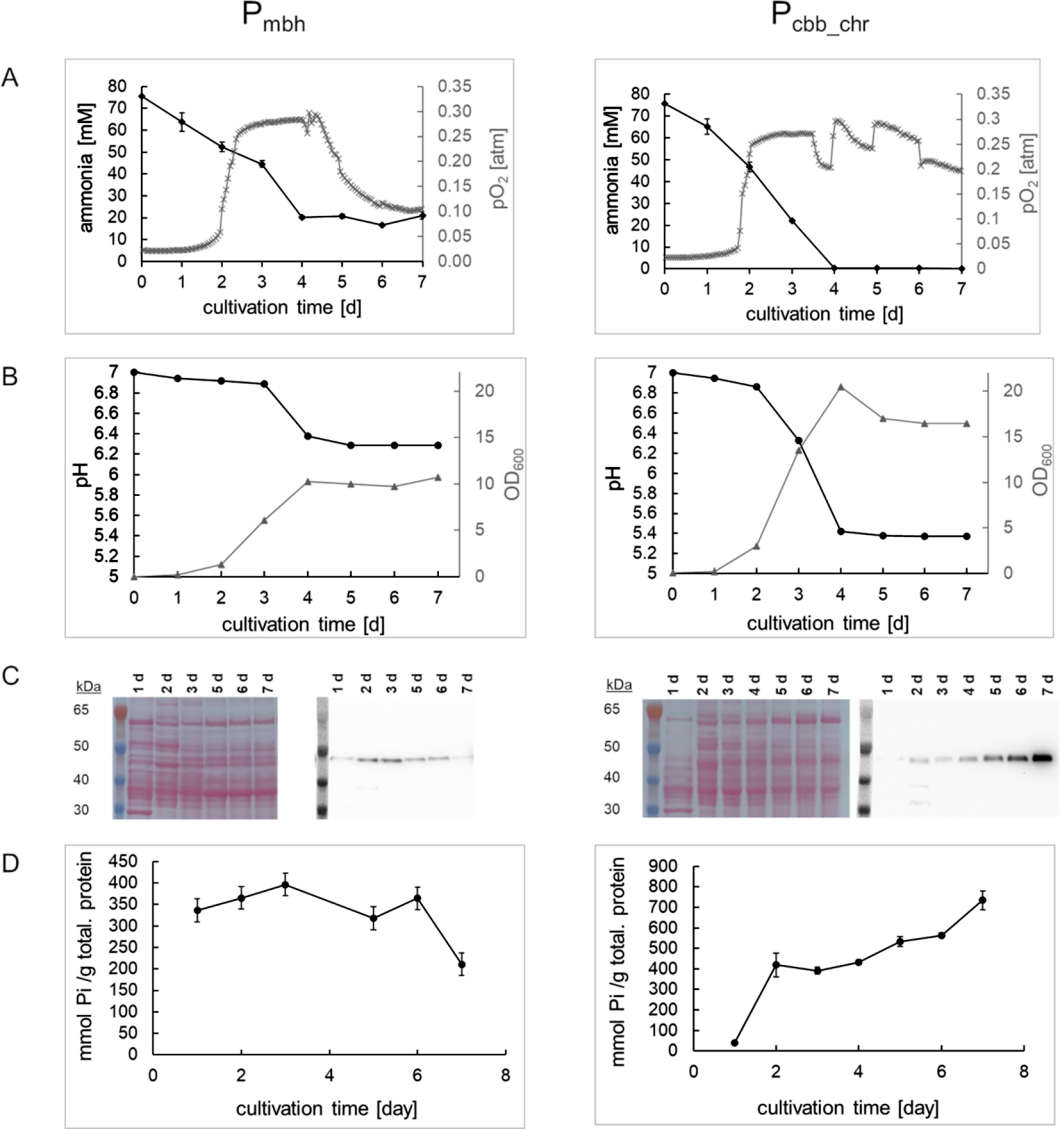
Chemolithoautotrophic fermentations of AppA production strains of *C. necator* H16 PHB^-4^. AppA expression from the RepPar plasmid was driven either by the membrane bound hydrogenase (P_mbh_) or the chromosomal Calvin-Benson-Bassham (P_cbb_chr_.) promoter. Cells were cultivated in optimized mineral media without any antibiotics using a lab-scale fermenter. (**A**) During fermentation of the P_mhb_ and the P_cbb_chr_ strains, oxygen partial pressure in the gas feed was adjusted to maintain 2% dissolved oxygen in the media. The consumption of ammonia as nitrogen source was monitored by daily sampling and an enzymatic assay. (**B**) Cell growth and the associated acidification of the culture media were observed by OD_600_ and pH measurements of daily samples taken for both strains, P_mbh_ and P_cbb_chr_, during the fermentation process. (**C**) AppA production was determined by immunoblot with an His-tag specific antibody for each cultivation day. As loading control, the PonceauS staining of the corresponding membrane is shown. (**D**) AppA activities were evaluated by measuring the phosphate released from phytic acid (15 min, 37 °C), due to the addition of cell lysate from the P_mbh_ and the P_cbb_chr_ strain

First, we tested the strain expressing *appA* under the control of the mbh promoter. During the first 24 h of fermentation, the strain showed a low oxygen demand and very little growth. After 24 h, the oxygen demand started to rise and the OD_600_ measurement indicated a shift from adaptation to growth phase (Fig. 4, A). Oxygen demand increased further from day two until day three, which correlated with an increase in biomass (Fig. 4, B). According to the OD_600_ measurements, cell growth stopped at day four of the fermentation, and a final cell dry weight (CDW) of 5 g/l could be reached. In theory, the used media composition would allow a maximum CDW of 8 g/l [38]. Yet, the *mbh-appA* strain stopped growth and consumption of the nitrogen source early, even though approximately one fourth of the initial ammonia was still present in the medium (Fig. 4, A). Measurements of the pH indicated a final pH of ~ 6.3, which was reached on day 4 of the fermentation (Fig. 4, B). When cells entered the stationary phase between day 4 and 5, oxygen consumption started to decline until the end of the fermentation. Highest AppA production levels and activities were found in cells harvested on day three (Fig. 4, C and D). All samples taken at later timepoints showed lower protein levels and activities, most likely due to protein degradation. The highest activity detected in bioreactor cultivations only yielded 54% of the activity per total protein found for the same strain cultivated in anaerobia pots.

Next, cells expressing *appA* from the chromosomal CBB promoter were tested in the gas fermenter. In the screening, this promoter achieved the second highest AppA production (Fig. 3, A–D) and indicated strong activation under autotrophic conditions. We hypothesized that this specificity might allow to decouple the growth from the production phase, and thereby possibly lead to higher AppA titers without any additional need for inducers which would complicate process control and add to the process costs. After a 24 h lag phase, cells started to grow exponentially (Fig. 4, B), and started to consume oxygen efficiently. Cells reached a final biomass of 8 g CDW/l, which is the theoretically possible maximum based on the nitrogen input. The pH in the fermentation broth finally decreased to 5.4 at day four when cells stopped to proliferate (Fig. 4, D). In contrast to cells expressing *app*A from the mbh promoter, expression with the chromosomal CBB promoters led to a steadily increasing production of AppA until day 7 of fermentation (Fig. 4, F). The phosphate release by AppA in the corresponding cell lysates showed the same trend (Fig. 4, H). The maximum AppA activity found at day seven led to approximately 750 mmol of phosphate per g total protein released after 15 min of reaction time, which is twice of the amount found for AppA produced from the mbh promoter.

For both fermentations, AppA activities were determined in the cell lysate for a time point that exhibited high biomass accumulation and AppA production. To achieve representative values for AppA activity, three reactions with cell lysate were run for 35 min and the released phosphate was determined every 5 min. The corresponding kinetic curves were used for calculation of AppA activity, and one unit was defined as one µmol phosphate released per minute. For the fermentation with AppA under the control of the mbh promoter, activity was determined with samples from day three, and with the CBB promoter at day seven, respectively. Due to the significantly higher biomass and the higher AppA production found with the CBB promoter, a 1.5 times higher yield in respect to the CDW and twice the product titer were found in comparison to the mbh promoter driven production (Table 1). For both the mbh and the chromosomal CBB promoter containing plasmids, 98–100% of the cells still grew on selection plates, containing kanamycin, after 7 days of gas fermentation (Table 1).

**Table 1:** Determination of AppA activity and the corresponding yields and titers achieved during gas fermentation

	Yield [U/mg CDW]	Titer [U/ml broth]	Plasmid stability [%]
P_mbh_	1.8 ± 0.2	9.9 ± 0.9	99 ± 1
P_cbb_chr_.	2.8 ± 0.3	22.3 ± 2.4	99 ± 1

## Discussion

Given its autotrophic lifestyle, lack of known pathogenicity, and high protein content, *C. necator* emerges as an intriguing candidate for enzyme production, particularly for use as a feed additive. However, the lack of efficient vectors for rapid gene insertion and stable antibiotic-free gene expression in *C. necator* poses a significant challenge. To address this gap, the present study focuses on the development of stable episomal plasmids that can be readily introduced into *C. necator* through electroporation. Our objective was to achieve stable protein production by ensuring high plasmid stability, which mostly depends on the choice of replicon and the implementation of a selection mechanism. Historically, most replicons employed on plasmids designed for electroporation in *C. necator* have exhibited notable stability challenges during extended cultivation periods [7, 9]. As our findings have substantiated, the RP4 partitioning system effectively mitigates the segregational instability associated with the high-copy-number pBBR1 replicon. This translates into an excellent plasmid stability of almost 100% over the course of a seven day gas fermentation (Table 1). This confirms that the reduction and slimming of the expression plasmids for improved electroporation did not affect the stabilizing role of the RP4 partitioning system. Alternative strategies that were shown to improve the stability of plasmids rely on post segregational killing by toxin antitoxin (TA) systems (reviewed in [39]). For example, stabilization of a conjugational plasmid was tested in a PHB negative *C. necator* strain applying the so-called hok/sok system [40, 41]. During fed batch cultivations, stability of the pBBR1-based plasmid – which produced eGFP – benefitted from the hok/sok system [40]. Yet, the stabilizing effects were not as strong as reported for the RP4 partitioning system ([12], this study), and even completely lost when cells were additionally stressed by isopropanol production [41].

Most promoter screenings published so far focused on heterologous or synthetic constitutive promoters [12, 30, 31]. To be able to thrive in an environment rich in H_2_, O_2_, and CO_2_, *C. necator* exhibits a distinctive array of enzymes arranged within several operons that are tightly regulated at the transcriptional level [28, 29]. Assuming that the respective promoters must be strong, and even more active on CO_2_ than on fructose, we tested the native hydrogenase and CBB promoters. When cells were grown on fructose, *appA* expression levels were found to be similar for the two hydrogenase (mbh and sh) and the constitutive j5 promoters (Fig. 3). Previous studies of the hydrogenase promoters reported intermediate activation on fructose, and strong activation on CO_2_/H_2_/O_2_ gas mixtures [29, 42]. When we compared AppA activities of cells expressing *app*A from these promoters under heterotrophic and chemolithotrophic conditions, no significant differences (p > 0.05) were observed (Fig. 3). The pBBR1 replicon used in our study was reported to be maintained at medium copy number per cell [9, 10] whereas the other studies used low copy number plasmids. Due to the complex and self-contained nature of the hydrogenase regulatory network (reviewed in [43]), it is possible that the presence of multiple copies of the mbh promoter somehow interferes with the native transcriptional regulation, thereby causing less pronounced repression on fructose. The low basal activation of the CBB promoter found on fructose was observed before and fits to reports of *C. necator* recycling the CO_2_ generated during fructose metabolism [44, 45].

Surprisingly, the frc promotor, tested for the first time in heterologous protein expression, showed no significant activation on fructose compared to lithotrophic conditions, as would be assumed due to the function of the downstream ORFs in fructose metabolism and transport [34]. As second novel promotor, phc also yielded rather weak *appA* expression levels and lower activation under chemolithotrophic conditions (p = 0.01). The corresponding phc-operon shows high similarities to a cell density sensing system described in *Ralstonia sonlanacearum*, and the corresponding promoter was therefore considered as interesting candidate in our screening [33]. For both promoters, more elaborate studies would be necessary to investigate in more detail, why expression levels were so low, but we can conclude, that these specific promoter sequences are rather unsuitable for high-level gene expression.

One challenge we faced during gas fermentations was the fact that cells expressing *appA* from the mbh promoter yielded comparably low biomass (Fig. 4). A possible explanation could be the metabolic burden posed by the strong overexpression of the heterologous gene. However, the lowered oxygen consumption observed during fermentation suggests another, more fundamental issue. Transcription of the mbh promoter is mainly dependent on the alternate, less abundant sigma factor σ^54^ and regulated by the positive transcription factor HoxA [29]. The presence of multiple copies of the mbh promoter on high copy number plasmids may lead to a competition for the corresponding sigma and transcription factors, and thereby limit production of the endogenous membrane bound hydrogenase. Since hydrogenases are essential for growth under chemolithotrophic conditions [46, 47] this could potentially impact the overall energy metabolism explaining the observed growth defect (Fig. 4, B) and decrease in AppA productivity in the last days of fermentation (Fig. 4, D). Gas transfer rates are expected to be rather low in anaerobia pots, which is why such an effect could play a bigger role in gas fermentations where high k_*L*_*a* values have been observed. The CBB promoters, in contrast, are dependent on the main bacterial sigma factor σ^70^, and regulated by CbbR and RegA [44, 48]. It has been consistently observed that the abundance of σ^70^ is higher compared to that of σ^54^ [49, 50] which makes any depletion of the σ^70^ factor less likely. Using the cbb_chr promoter, AppA production even increased after the maximum biomass had been reached (Fig. 4). *C. necator* accumulates PHB on CO_2_ under nitrogen limiting conditions [32], which suggests ongoing activation of CBB pathway genes (including cbb_chr) even in stationary cells.

Phytases, like AppA, are among the most important enzymes for the feed industry. AppA is mainly produced in organisms like *E. coli* or *Pichia pastoris*, for which production procedures are established. Due to the efficient secretion system, the available strong promoters and the high cell densities achieved with *P. pastori*s, the highest AppA titer reported was approximately 5.000 U/ml in this host [26]. This is considerably higher than anything reported for bacterial systems. By applying the *E. coli* BL21 strain, 100 U/ml were reached in a fed batch fermentation, which yielded a similar biomass as we reached in the gas fermentations described herein [25]. Phytase production was also attempted in generally recognized as safe (GRAS) bacteria. For example, expression of *appA* in *Streptomyces rimosus* or *Lactococcus lactis* resulted in 5 U/ml and 20 U/ml, respectively [51, 52]. In this study, we obtained 22 U/ml (2.8 U/mg CDW) of AppA activity without any antibiotic pressure. Another, unique feature of phytase production in *C. necator* is the use of CO_2_ as abundant carbon source. The biomass generated in our gas fermentation consists of approximately 14 g of assimilated CO_2_, which correlates to CO_2_ capture rates of 146 mg l^− 1^ h^− 1^. As discussed, 500 U of phytase per kg of feed are recommended for chicken and poultry [23, 24]. Irrespective of any activity loss during a potential downstream processing of the *C. necator* biomass, this would correspond to ~ 179 mg dried cells from lab-scale fermentation of our best AppA production strain. In 2021, EFSA authorized *C. necator* for the production of food additives, flavorings and food enzymes on the European market [53]. Indeed, studies testing *C. necator* H16 PHB^− 4^ as animal feed were already conducted [20, 54] and none of them reported any toxic ingredients in the prepared biomass. Since no pathogenic strains are known, a direct feed of *C. necator* biomass containing AppA to pig or poultry may be conceivable in the future.

## Conclusion

Electroporation of *C. necator* H16 is becoming the gene delivery strategy of choice. However, most replicons used on electroporation vectors were either unstable or of low copy number [7–9] which is suboptimal for heterologous protein expression. The slim pBBR1 replicon-based, high copy number plasmids tested in this study are filling this niche and showed stable maintenance in gas fermentations. Due to the fast and easy delivery of the plasmid, it was easily possible to screen multiple native promoters for production of phytase AppA*Ec*. These experiments indicated superior performance of native CBB, and hydrogenase promoters compared to the constitutive j5 promoter during lithoautotrophic growth. The best performing promoter, found upstream of the chromosomal CBB operon, led to 22 U/ml phytase activity in gas fermentations. Even though high-level AppA production has previously been achieved using *P. pastoris* and *E. coli* [25, 26], *C. necator* offers the clear advantage to be grown on antibiotic-free mineral media with CO_2_ as a carbon source. The plasmids presented in this study can easily be tested for other interesting phytase candidates and enzymes in future. Moreover, considering the increasing interest in *C. necator* as a fundamental player in modern green biotechnology, coupled with the yet-to-be-explored potential for engineering strain backgrounds, it is plausible to anticipate further advancements in the field of heterologous protein expression in the near future.

## Materials and methods

### Strains

Plasmid construction was performed with *E. coli* Top10 (Invitrogen). Initial characterization of new constructs was performed in *C. necator* H16 (DSM 428). For phytase expression, the *C. necator* H16 PHB^− 4^ (DSM 541) strain was used. A detailed list of the used strains can be found in Additional file 1 (Table S1).

### Cloning and plasmid delivery

Plasmids constructed in this study were based on previously published vectors for conjugation as gene delivery method [12]. Gibson assembly was used to generate the new plasmids [55]. PCRs were performed with the Q5 polymerase from NEB. To enable efficient electroporation, plasmid sizes were decreased by removal of the RP4 mobility region (mob). Native *C. necator* promoters were amplified by PCR from *C. necator* H16 genomic DNA, *appA* was amplified from *E. coli* K12 substr. MG1655 (NP_415500.1) and introduced into the mob free plasmids. Cloned plasmids were verified by restriction analysis and Sanger sequencing. Plasmids and primers used, are listed in Tables S2 and S3 of the Additional file 1. Identification of the *C. necator* H16 genome regions used as promoters are listed in Table S4.

Electrocompetent *C. necator* cells were prepared as described in Taghavi et al. [56]. with slight modifications. Briefly, 200 ml of SOB media (5 g/l yeast extract, 20 g/l tryptone, 0.6 g/l NaCl, 0.2 g/l KCl) were inoculated to an OD_600_ of 0.1 and cultivated in 1 l unbaffled shake flasks (28 °C, 170 rpm) until cells reached an OD_600_ of 0.6–0.8. Cultures were incubated on ice for 30 min, split in 8 parts and harvested by centrifugation (3200 g, 10 min, 4 °C). Each cell pellet was washed three times with ice cold 15% glycerol: The first washing step was performed with 25 ml, the second with 15 ml, and the third with 5 ml of 15% glycerol. After the final washing step, the *C. necator* cell pellets were resuspended in 15% glycerol. A cell pellet corresponding to a 200 ml culture with a measured O_600_ of 0.6 was resuspended in 0.9 ml of the prepared glycerol solution. If harvested at higher cell densities, the volume was adjusted accordingly. Aliquots of 50 µl were shock frozen with liquid nitrogen and stored at -80 °C.

Electroporation of *C. necator* was performed with a MicroPulser electroporator (Bio-Rad) in 2 mm cuvettes with 2.5 kV (12.5 kV/cm) for approximately 5.8 ms. As a standard 50 ng of plasmid were transformed. Cells were regenerated in 1 ml SOC media (20 g/l tryptone, 5 g/l yeast extract, 0.58 g/l NaCl, 2 g/l MgCl_2_*6H_2_O, 0.186 g/l KCl, 2.46 g/l MgSO_4_*7H_2_O, 3.96 g/l glucose-monohydrate) for 2 h at 30 °C before plating on tryptic soy broth (TSB) supplemented with 200 µg/ml kanamycin.

### Plasmid stability

Segregational plasmid stability in *C. necator* was evaluated on TSB as reported previously [12]. In summary, *C. necator* containing the target plasmid was cultivated overnight in TSB containing 200 µg/ml kanamycin. Main cultures w/o antibiotics were inoculated to an OD_600_ of 0.2 and cultivated for 24 h. This culture then served as overnight culture to inoculate another main culture to an OD_600_ of 0.2 that was subsequently cultivated for 24 h. This step was repeated four times. To check for stability of the plasmids during each cultivation cycle, cultivation broth corresponding to approximately 50 cfu was plated on TSB plates. The freshly formed colonies were counted and visually inspected for the presence of eGFP. Rates of plasmid loss were additionally determined by transfer of colonies to TSB plates with kanamycin. Experiments were performed in biological triplicates.

Growth rates and fluorescence achieved with the four eGFP expression plasmids constructed in this study were determined during growth on TSB w/o antibiotic. Main cultures were inoculated from kanamycin containing precultures to OD_600_ 0.1. Fluorescence was measured with Synergy MX microplate reader (BioTek, 480/509 nm ex/em) in a 2 h interval for 12 h, together with OD_600_. Cultivations were performed with three independent transformants of each plasmid.

### Promoter screening

Screening of promoters for AppA*Ec* expression in *C. necator* was performed on mineral media [36] adapted for increased biomass accumulation (6 g/l KH_2_PO_4_, 18 g/l Na_2_HPO_4_*2H_2_O, 10 g/l (NH_4_)_2_SO_4_, 0.2 g/l MgSO_4_*7H_2_O, 0.05 g/l NH_4_Fe(III)citrate, 0.02 g/l CaCl_2_*2H_2_O, 0.06 mg/l Na_2_WO_4_*2H_2_O, 0.6 mg/l H_3_BO_3_, 0.4 mg/l CoCl_2_*6H_2_O, 0.2 mg/l ZnSO_4_*7H_2_O, 0.06 mg/l MnCl_2_*4H_2_O, 0.06 mg/l NaMoO_4_*2H_2_O, 0.4 mg/l NiCl_2_*6H_2_O, 0.02 mg/l CuSO_4_*7H_2_O). Cultivations were performed in 100 ml baffled shake flasks containing 20 ml of mineral media (28 °C, 120 rpm) supplemented with kanamycin (200 µg/ml). For heterotrophic cultivations, 20 g/l fructose was used as carbon source. Autotroph cultivations were performed by applying an atmosphere of 11% oxygen, 11% carbon dioxide, 67% hydrogen and 11% air in an anaerobia pot (5.8 l) containing up to 6 shake flasks. Fresh atmosphere was applied when the pressure in the anaerobia pot decreased by 30%. Cultures were inoculated to OD_600_ 0.2 from mineral media overnight cultures containing fructose. Heterotrophic cultures were harvested after 72 h, when cell growth stopped completely. Autotrophic cultivations were run for 5 days and harvested when no further depletion of the gas atmosphere was detectable.

Cell dry weight of the different strains in the screening was determined by harvesting of 10 ml culture, washing of the cell pellet with double-distilled water and drying of the corresponding pellet for 24 h at 60 °C.

### Gas fermentation

Autotrophic precultures were cultivated in 300 ml baffled shake flasks containing 50 ml of optimized mineral media (4.5 g/l KH_2_PO_4_, 13.5 g/l Na_2_HPO_4_*2H_2_O, 5 g/l (NH_4_)_2_SO_4_, 0.4 g/l MgSO_4_*7H_2_O, 0.05 g/l NH_4_Fe(III)citrate, 0.02 g/l CaCl_2_*2H_2_O, 0.06 mg/l Na_2_WO_4_*2H_2_O, 0.6 mg/l H_3_BO_3_, 0.4 mg/l CoCl_2_*6H_2_O, 0.2 mg/l ZnSO_4_*7H_2_O, 0.06 mg/l MnCl_2_*4H_2_O, 0.06 mg/l NaMoO_4_*2H_2_O, 0.4 mg/l NiCl_2_*6H_2_O, 0.02 mg/l CuSO_4_*7H_2_O) supplemented with kanamycin (200 µg/ml) for 3–4 days (28 °C,120 rpm). A 11% oxygen, 11% carbon dioxide, 67% hydrogen and 11% air atmosphere was maintained by usage of an anaerobia pot. As the cultivation vessel for main cultures, a stirred 1000 mL DURAN® GLS 80 wide-neck threaded glass bottle was used. Explosion-proof stirring was accomplished by a magnetic anchor stirrer (stirrer reactor cap GLS 80, from DWK Life Sciences purchased at CarlRoth) driven by a magnetic stirrer atexMIXdrive at 240 rpm (2 mag AG) [36]. The empty reactor was sterilized by autoclaving, while the oxygen probe was disinfected with 70% EtOH prior to use. The media components were combined under a laminar flow. The double-walled reactor (ETP3.1, Lactan**)** was kept at a constant temperature of 30 °C using an external water bath. The oxygen probe was calibrated by two-point calibration prior to fermentation. For this purpose, the mineral medium was purged with nitrogen (0% O_2_) at constant temperature (30 °C). Point 2 (100% O_2_) was obtained by purging the reactor with pure oxygen [36].

Before starting the gas fermentation, the optimized mineral media was equilibrated with a gas mixture of 2:5:93 (O_2_:CO_2_:H_2_) for 30 min. Afterwards, the fermenter was inoculated to 0.025–0.06 OD_600_. During fermentation, dissolved oxygen (2% $\cong$ 0.75 mg/l) concentration was automatically controlled as recently described, while CO_2_ supply was kept constant at 5% (v/v, in the gas feed) [37]. Hydrogen was added to a total gas flow of 400 ml/min. Sampling was done after stopping the potentially explosive gas mixture via remote connection and purging the reactor with nitrogen for 5 min. Samples were taken with a 120 mm long needle connected to a syringe and analyzed further outside the gas laboratory. To avoid foaming, PPG (polypropylene glycol) was added after 1 day of fermentation.

Gases (H_2_, O_2_, N_2_, CO_2_) for all autotrophic cultivations were purchased from Air Liquide Austria at purities of 5.0 (99.999%) for O_2_, N_2_ and H_2_ and 2.5 (99.5%) for CO_2_. Polypropylene glycol P 2,000 (PPG) was purchased at Sigma Aldrich. All components of the mineral media were purchased from CarlRoth.

The remaining ammonia in the fermentation broth was determined by the Ammonia Assay Kit (Rapid) from Megazyme (K-AMIAR). The assay was performed in microplates corresponding to the manufacturer’s instructions.

Stability of the two episomal plasmids tested during fermentations was evaluated by plating of broth from day seven on TSB agar and re-streaking of 50 single colonies on plates containing kanamycin.

Cell dry weight was determined by drying of a cell pellet, washed with double-distilled water, corresponding to 50 ml of culture broth for 24 h at 60 °C.

### Immunoblot analysis

Cell lysis was performed with the BugBuster Protein Extraction Reagent (Novagen, Merck). The reagent concentrate was diluted with 100 mM Tris-HCl buffer (pH 8). Protein concentrations were measured with the Pierce™ BCA Protein Assay Kit (Thermo Fisher Scientific).

SDS-PAGE samples containing 10 µg protein were separated on 4–12% Bis-Tris gels (NuPAGE, Thermo Fisher Scientific). Blotting on a nitrocellulose membrane was achieved via electric current. AppA was detected via a conjugated mouse anti-His-HRP antibody (1:1000, 27E8, Cell Signaling Technology). For signal detection, the Clarity Max Western ECL substrate (Bio-Rad) was used in combination with the Syngene® GBox HR16.

### Phytase activity assay

Standard AppA reactions were performed in acetate buffer (0.1 M, pH 4.5) at 37 °C. As substrate, sodium phytate was added (8 mM). Reactions were started by addition of cell free extracts obtained from BugBuster Protein Extractions and stopped by acidification (0.5 M HCl). In total, two samples were taken for each reaction: One directly after mixing of the reaction and one after 15 min to determine phosphate release by AppA. Activity of cell lysates deriving from the gas fermentation were determined by measuring phosphate release over 35 min in a 5 min interval.

Phosphate content of the samples was determined by the Saheki method [57]. Briefly, 120 µl of freshly prepared Saheki solution (12 mM ammonium molybdate, 80 mM zinc acetate and 2% ascorbic acid, pH 5) were added to 10 µl of sample volume. After 15 min of incubation at room temperature, absorbance at 850 nm was measured on an Eon microplate spectrophotometer (BioTek). For calibration, potassium dihydrogen phosphate solutions (2 mM-77 µM) were used. All assay solutions were prepared with highly pure water (max. 18.2 MΩ cm) to minimize background caused by abundant phosphate. Activities were always determined in technical triplicates.

### Statistical analysis

To determine significance of observed difference in experimental results a to tailed Student’s T-test was used to calculated corresponding p-values.

### Electronic supplementary material

Below is the link to the electronic supplementary material.

Supplementary Material 1

## Data Availability

All relevant data are within the manuscript and its Supporting Information file. The expression plasmids were deposited to Addgene.
