# Supplementary material for: CO2-based production of phytase from highly stable expression plasmids in Cupriavidus necator H16
Source: Microb Cell Fact. 2024 Jan 3;23:9. doi: 10.1186/s12934-023-02280-2 (PMC10763379; doi:10.1186/s12934-023-02280-2)
Supplement: Supplementary file 1 — Supplementary Material 1 [file 12934_2023_2280_MOESM1_ESM.docx]

## Additional material


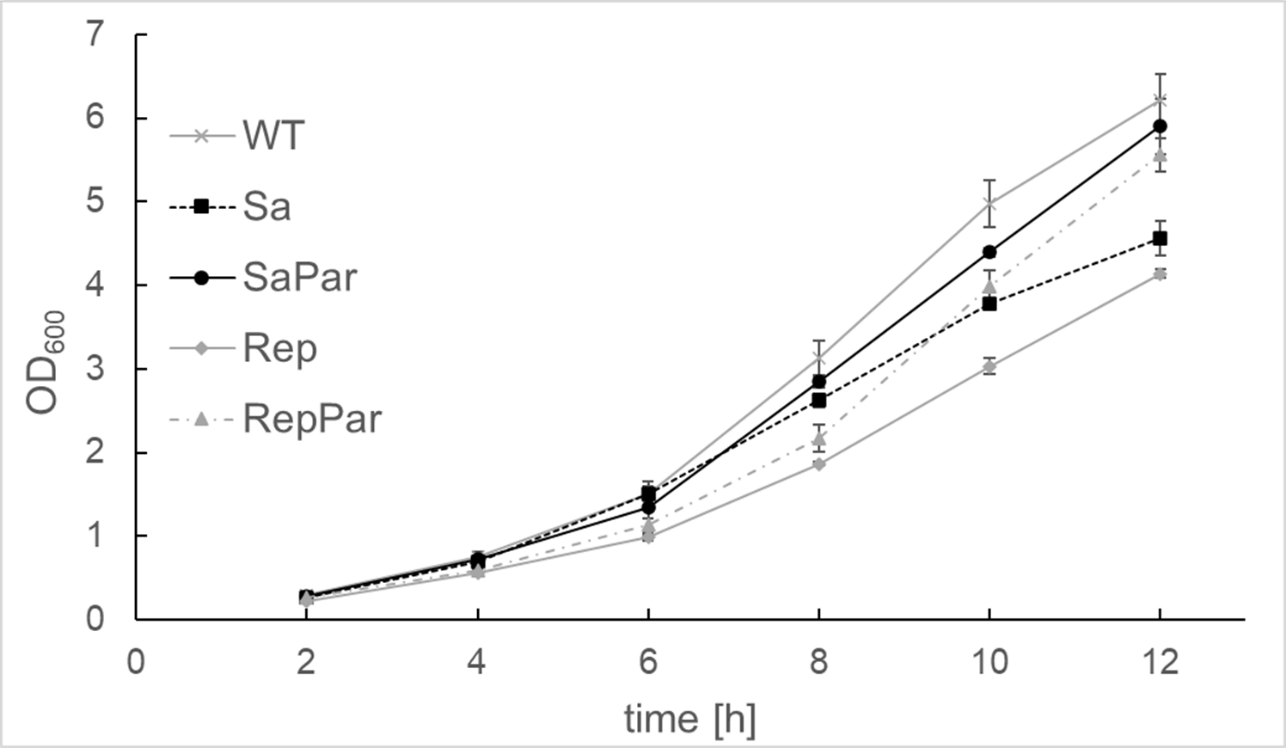


*Figure S1 Measurement of cell growth during a 12 h cultivation of C. necator H16 carrying the four constructed plasmids (n=3). As control wild type C. necator H16 carrying no plasmid (WT) was added to the cultivation. Cultures grew on antibiotic free TSB media at 28°C as described in Materials and Methods. Error bars, SEM*

To estimate the influence of the chosen promoters on biomass production, cell dry weights (CDW) were determined by a single measurement**.** On fructose supplemented media, 7-8 g CDW/l media were obtained with all, but the mbh and the j5 promoters (**Figure S2)**. While strains producing AppA from the mbh promoter still reached 6.3 g CDW/l, utilization of the j5 promoter decreased the final biomass to 2.6 g CDW/l. Under autotrophic conditions, an even stronger effect on the final biomass (**Figur S2**) was observed, which ranged from 0.5-4.5 g CDW/l, whereas the j5 promoter again yielded the lowest biomass accumulation.


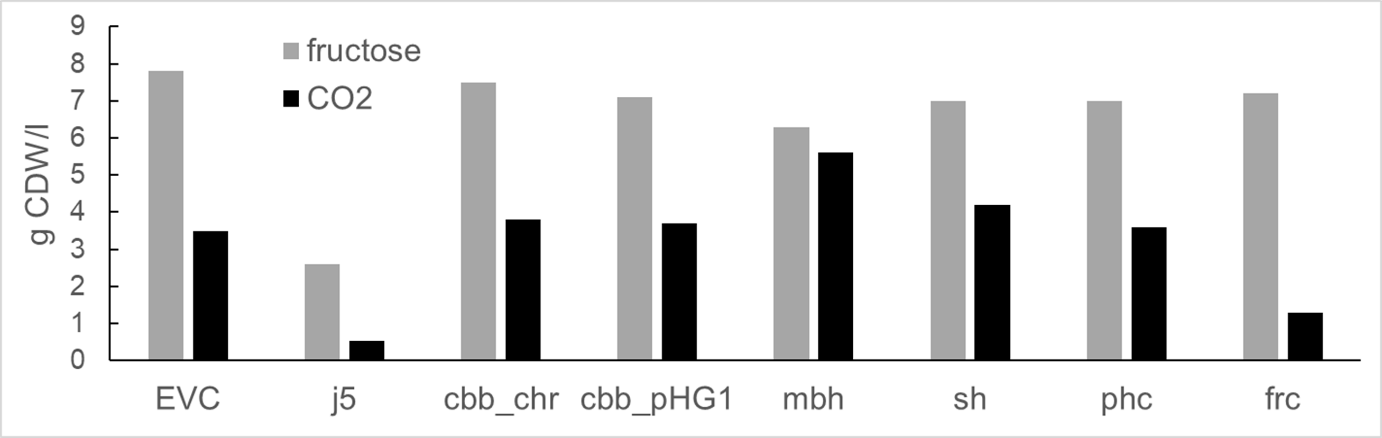


*Figure S2 Final cell dry weight (CDW) was determined for each cultivation in the promotor screening to monitor the influence of the used AppA expression system on biomass accumulation. C. necator strains were cultivated in mineral media supplemented with kanamycin until stationary phase was reached. Fructose was used as a carbon source under heterotrophic conditions. To facilitate growth under chemolithoautotrophic conditions, cultivations were performed in an anaerobia pot containing a CO_2_/H_2_/O_2_ atmosphere.*

Table S1: List of strains used in this study.

| **Strain** | **Description** | **Source** |
| --- | --- | --- |
| *E. coli* TOP10 | Cloning strain | Invitrogen (C404010) |
| *E. coli* K-12 MG1655 | PCR amplification of AppA | DSMZ (DSM 18039) |
| *Cupriavidus necator* H16 | wild type strain | DSMZ (DSM 428) |
| *Cupriavidus necator* H16 PHB^-4^ | polyhydroxybutyrate deficient strain, used for AppA production | DSMZ (DSM 541) |

Table S2: Plasmids used in this work.

| **Plasmid** | **Description** | **Source** |
| --- | --- | --- |
| pKSa-Ptac-egfp | Km^r^, P_tac_, eGFP, pSa origin of replication, partition region par and mobilization sequence mob from the RP4 plasmid | (1) |
| pKRep-Ptac-egfp | Km^r^, P_tac_, eGFP, par, RP4mob, pBBR1 origin of replication | (1) |
| pKRSF1010-P_T5_-egfp | Km^r^, P_T5_, eGFP, par, RSF1010 mob and origin of replication | (1) |
| pKESa | Km^r^, P_T5_, eGFP, pSa origin of replication | This work |
| pKESaPar | Km^r^, P_T5_, eGFP, pSa origin of replication, par | This work |
| pKERep | Km^r^, P_T5_, eGFP, pBBR1 origin of replication | This work |
| pKERepPar | Km^r^, P_T5_, eGFP, pBBR1 origin of replication, par | This work |
| pKPRepPar_P_j5_-EVC | Km^r^, P_j5_, random stuffer sequence, pBBR1 origin of replication, par | This work |
| pKPRepPar_P_j5_ | Km^r^, P_j5_, *E. coli* AppA, pBBR1 origin of replication, par | This work |
| pKPRepPar_P_cbb_chr._ | Km^r^, P_cbb_chr._, *E. coli* AppA, pBBR1 origin of replication, par | This work |
| pKPRepPar_P_cbb_pHG1_ | Km^r^, P_cbb_pHG1_, *E. coli* AppA, pBBR1 origin of replication, par | This work |
| pKPRepPar_P_mbh_ | Km^r^, P_mbh_, *E. coli* AppA, pBBR1 origin of replication, par | This work |
| pKPRepPar_P_sh_ | Km^r^, P_sh_, *E. coli* AppA, pBBR1 origin of replication, par | This work |
| pKPRepPar_P_phc_ | Km^r^, P_phc_, *E. coli* AppA, pBBR1 origin of replication, par | This work |
| pKPRepPar_P_frc_ | Km^r^, P_frc_, *E. coli* AppA, pBBR1 origin of replication, par | This work |

Table S3: Primer used in this work. Overhangs for Gibson assembly, lowercase letters; Primer part for initial binding at target, underlined uppercase letters; Added His-Tag, written in bold.

| pCR-pSa-ori+_f1 | cgtgagttttcgttccactgagcgtcagacactagtGCTACTTTCCGAACGACTCCTG |
| --- | --- |
| pCR-pSa-ori+_r1 | gggcagtgcccggcttttctcaccttttcgcctgcagGCAGACCAGAACCAATCCTATTC |
| pCR-pBBRori+_f1 | cgtgagttttcgttccactgagcgtcagacactagtGCGGCCACCGGCTGGC |
| pCR-pBBRori+_r1 | ggcagtgcccggcttttctcaccttttcgcctgcagCTACCGGCGCGGCAGCG |
| pHyb-pKERep-par-neg_f1 | agccgccgacacgggtcacgctgccgcgccggtagctgcagcccaaatcataaaaaatttatttgctttgtgagcg |
| pHyb-pKERep-par-neg_r1 | cgctcacaaagcaaataaattttttatgatttgggctgcagctaccggcgcggcagcgtgacccgtgtcggcggct |
| pCR-pKESa-par-neg_f1 | GtgctgaataggattggttctggtctgcctgcagggccgccaaatcataaaaaatTTATTTGCTTTGTGAGCGGATAACAATTATAATAG |
| pCR-pKESa-par-neg_r1 | caaaattatttctagaggatccccgggtaccgagctcgaattCTATTATAATTGTTATCCGCTCACAAAGCAAATAA |
| pCR-pKPRep-Pj5_f1 | ccgctacgggctttttcatgccctgcccgcggccgcaaaaaccgttattgacaCAGGTGGAAATTTAGAATATACTGAAGC |
| pCR-pKPRep-Pj5_r1 | tcgctttcatggatcctatatctccttcttaaagttaaacaaaaaGCTTCAGTATATTCTAAATTTCCACCTG |
| pCR-pKPRep-EcAppA_f1 | gtttaactttaagaaggagatataggatccATGAAAGCGATCTTAATCCCATTTTTATC |
| pCR-pKPRep-EcAppA_r1 | gaaaatcttctctcatccgccaaaacagccgaattc**ttagtgatggtgatggtgatg**CAAACTGCACGCCGGTATGC |
| pCR-Pcbb-chr_f1 | ccgctacgggctttttcatgccctgcccgcggccgctattaGGCGTGCCTGGCCACGG |
| pCR-Pcbb_r1 | agaagagataaaaatgggattaagatcgctttcatGCTTGTCTCCTTGCGTGGTTGAGC |
| pCR-Pcbb-pHG_f1 | ccgctacgggctttttcatgccctgcccgcggccgcttaatGCGGCGCGCACGAAGCTA |
| pCR-Pmbh_f1 | ccgctacgggctttttcatgccctgcccgcggccgcGTAGCTGCGTGGCATTGCTC |
| pCR-Pmbh_r1 | gaagagataaaaatgggattaagatcgctttcatAACCTGTCTCCTAATTTCTGTATTGG |
| pCR-Psh_f1 | ccgctacgggctttttcatgccctgcccgcggccgcCTGCCTCCGGTCACCCG |
| pCR-Psh_r1 | gaagagataaaaatgggattaagatcgctttcatGTTGTCTCCTCCTTACTAATGTTCGC |
| pCR-Pphc_f1 | ccgctacgggctttttcatgccctgcccgcggccgcCTTGCCATCCAGGTGGAAGG |
| pCR-Pphc_r1 | agaagagataaaaatgggattaagatcgctttcatCACCAGGGACTTTCTCTGGAG |
| pCR-Pfrc_f1 | ccgctacgggctttttcatgccctgcccgcggccgcCGGGTACTTCACCATGCAGATG |
| pCR-Pfrc_r1 | agaagagataaaaatgggattaagatcgctttcatCTTGTCTCCGTCCGCTATTAATTAATTC |

Table S4: Genomic location of the used promoters.

|  | **coordinates** | **length** | **Accession** |
| --- | --- | --- | --- |
| P_cbb-chr_ | 1562930..1563165 | 236 bp | NZ_CP039288.1 |
| P_cbb-pHG_ | 450507..450728 | 222 bp | NZ_CP039289.1 |
| P_mbh_ | 451710..452139 | 544 bp | NZ_CP039289.1 |
| P_sh_ | 79365..79711 | 347 bp | NZ_CP039289.1 |
| P_phc_ | 3378456..3378942 | 487 bp | NZ_CP039287.1 |
| P_frc_ | 1681137..1681371 | 235 bp | NZ_CP039288.1 |

**Safety concept for gas fermentation**

Gas cultivations were performed in explosion safe environment according to Austrian and European safety standards (ATEX directive RL 1999/92/EG and RL 2014/34/EU). A detailed safety analysis of equipment used is described in more detail in Lambauer and Kratzer (2). Only persons trained by TÜV Austria were allowed to enter the laboratory and handle the reactor, wearing antistatic clothing and a hydrogen sensor for personal protection. The entire laboratory as well as the reactor and the sensor were grounded. An adapted schematic diagram of the gas laboratory can be found in **Figure S3**.


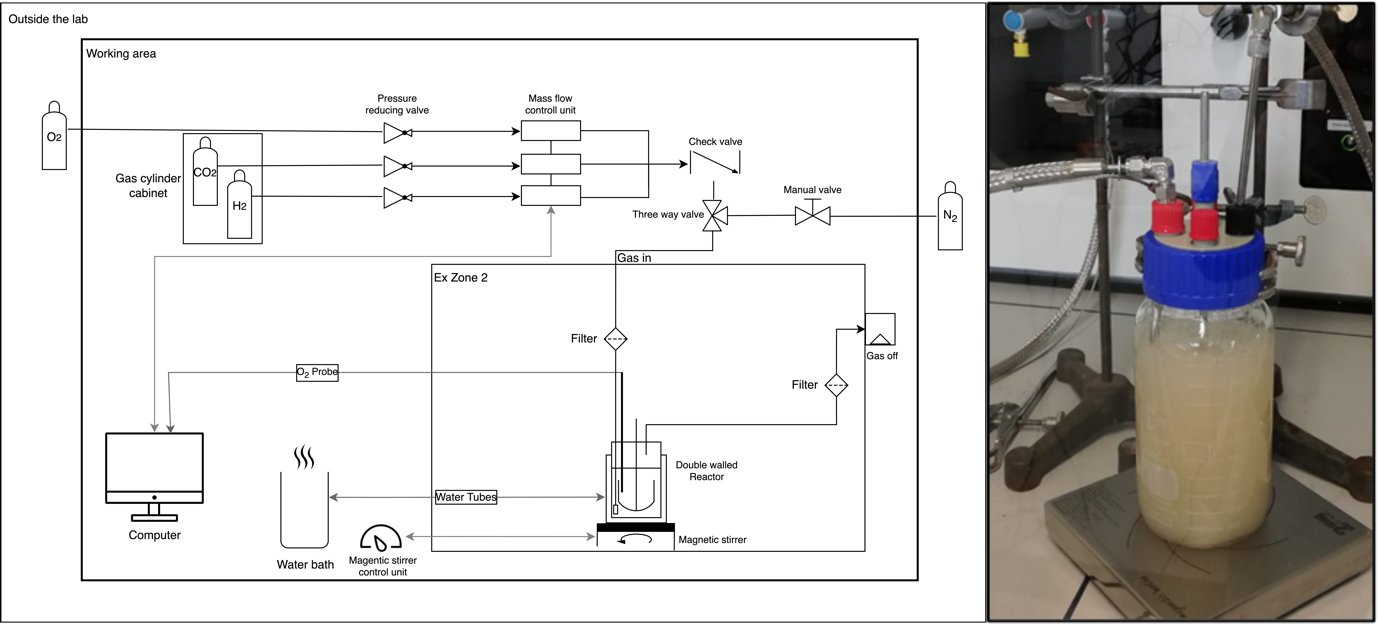


Figure S3 Scheme of gas fermentation laboratory. The gases were mixed statically and flow through a frit into the temperature-controlled fermentation broth. The composition of the gas mixture was automatically adjusted (using LabVIEW) depending on the measured dissolved oxygen concentration. Samples were taken by stopping the gas supply and purging the reactor with nitrogen. The exhaust gas is diluted directly in the explosion-proof fume hood (580 m^3^/h). The entire fermentation setup was grounded.

**>pKESa**

acatcctggggcacaagctggagtacaactacaacagccacaacgtctatatcatggccgacaagcagaagaacggcatcaaggtgaacttcaagatccgccacaacatcgaggacggcagcgtgcagctcgccgaccactaccagcagaacacccccatcggcgacggccccgtgctgctgcccgacaaccactacctgagcacccagtccgccctgagcaaagaccccaacgagaagcgcgatcacatggtcctgctggagttcgtgaccgccgccgggatcactctcggcatggacgagctgtacaagtaaaagcttggctgttttggcggatgagagaagattttcagcctgatacagattaaatcagaacgcagaagcggtctgataaaacagaatttgcctggcggcagtagcgcggtggtcccacctgaccccatgccgaactcagaagtgaaacgccgtagcgccgatggtagtgtggggtctccccatgcgagagtagggaactgccaggcatcaaataaaacgaaaggctcagtcgaaagactgggcctttcgttttatctgttgtttgtcggtgaacgctctcctgagtaggacaaatccgccgggagcggatttgaacgttgcgaagcaacggcccggagggtggcgggcaggacgcccgccataaactgccaggcatcaaattaagcagaaggccatcctgacggatggcctttttgcgtttctacaaactcttttgtttatttttctaaatacattcaaatatgtatccgctcatgaacaataaaactgtctgcttacataaacagtaatacaaggggtgttatgagccatattcaacgggaaacgtcttgctctaggccgcgattaaattccaacatggatgctgatttatatgggtataaatgggctcgcgataatgtcgggcaatcaggtgcgacaatctatcgattgtatgggaagcccgatgcgccagagttgtttctgaaacatggcaaaggtagcgttgccaatgatgttacagatgagatggtcagactaaactggctgacggaatttatgcctcttccgaccatcaagcattttatccgtactcctgatgatgcatggttactcaccactgcgatccccgggaaaacagcattccaggtattagaagaatatcctgattcaggtgaaaatattgttgatgcgctggcagtgttcctgcgccggttgcattcgattcctgtttgtaattgtccttttaacagcgatcgcgtatttcgtctcgctcaggcgcaatcacgaatgaataacggtttggttgatgcgagtgattttgatgacgagcgtaatggctggcctgttgaacaagtctggaaagaaatgcataaacttttgccattctcaccggattcagtcgtcactcatggtgatttctcacttgataaccttatttttgacgaggggaaattaataggttgtattgatgttggacgagtcggaatcgcagaccgataccaggatcttgccatcctatggaactgcctcggtgagttttctccttcattacagaaacggctttttcaaaaatatggtattgataatcctgatatgaataaattgcagtttcatttgatgctcgatgagtttttctaagaattaattcatgaccaaaatcccttaacgtgagttttcgttccactgagcgtcagacactagtgctactttccgaacgactcctgcgttgatcggaaatccagaagcccgagaggttgccgcctttcgggctttttctttttcaaaaaaaaaaatttataaaacgatctgttgcggccgccgggttgtgggcaaaggcgctcgacggtgggcaaccgcttgcggttgtccacgggcggagccggtgcgcgtagcgcattgtccacaagccaagggcgaccaataattgatatatatattcataattgaaaagctaattgaacatactacttgctgtaactacttgccggagcgaggggtgtttgcaagctgttgatctgaaagggctattagcgttctcacgtgcctttttgattagcgatttcacgtgaccttattagcgatttcacgtactccgattagcgatttcacgtaccctgattagcgatttcacgtggatagtttttggagcgggccggaaagccccgtgaatcaaggctttgcggggcattagcggtttcacgtggataactaccctctatccacaggcttccggggataaaaaagcccgctcgacggcgggctgttggatgggaaggcttgaccaagccaagcgtagcgttggcctggtcaagtcggaggggggccgatgcgagcgcccttgccgggtgcgcgggtgacatgcaggcgtgtggatttgatgcgcaggcattcgccgtcatcttcgatgcagtcgcttgcctcgggatagacaatcaacacttcgcgtaggcgctttttgaagttgtatttgaagctggcgagtgctgcccgctctgcccgctctcgggccttatcgtccagttcgggcgagttgcgtgcgcggctgccataggatgagccgaattgcgcttgcagggcgacccaagggatttgcacgaaggggcggcccttggcccgcaacaggaacacgcgataggtcagccacgtgtaaatgtccatcgcaagcggagactgccgcaaggcatgcaggtagtcgattcggataggaaccggtgagcgggtgacttcctcgaagaaatcgcctgtgagggtgagggtgctatcccatagcgcccgatcttctggccgcttgggattccagaatagaaaagcgcgcttggcaatgacgacgttctcaatgccgaagtcattgccttgctcgccggcaagcgaaatcatggatgaaaacaggcgttgcgcctgattgcgaagggtggccgtgtaacggccatcggtgtgcattccgagcctttgtagaaattccgattgcgaccggccaaggttcaacacggggtctttcgttcgcacggcctcggtgcatatccaagcaagcaaggtgcgcggcatagaaccgtagggcaggccgatgctcggcttgcccatgatcgacaaggtgacgatgccattggtgcgctcaaagtagctggtcttggggtcggtgtggggcatggtcgcttgcacaaggcaacgggccatgtagccgactaagccagcttcgcgggcatcctccatttcgagcgcgaggctcgtcttgatgatctcgttgatacgatggccgggggctttgttgttcttaggcatgttgttccctccccggcatggtgatggttggtctagtgtttgtgggtttgatgttccggcgtttgatgaacaggcgcaaggtgtgagggctgacgcctaacaactcggctgcgcgactttgcggcaagccaaggttcacgtatgcctgtacttcatcaatacggctgtccagcttcaaggcgctcgatttgctgcccttgggtcgcccgagcgtcttgccgcgctctctggcgacttgtagcgcctcggtggtacgtgcctgaatgaaatgccgctcgatctgtgcagccaagccaagcacggttgccatgatgtcgctttgtaggctgccgtccatgatgatcttctgtttggtcacatggacgattaggccgcgctcgctcgccgctttgagaatttccaaggcggcgagggcggaaccggcaatgcgcgtaatctccggcgtcagtagcacgtcgccacgctcggccttttcgatgattgctccgagcttgcgcttgcgccagtcctttgctctgctggcaatttcttcctcgatctgtagcggcgcgaagcctttggcgttcgcgtattcgagcaaaccgtatttttggttttccgggtcttggccgtcacgcgaaacccggagataggcatagtattttggcatttgcagggaaaacgtcagattcggttaaacatgcctcattctagcgcagattaaataggaattaaataccctgttgcggtatagataaaacgttggtttgttctgccctatgagcgtacaaaaaaggccgggtgagtggcccggccttcgtttaggtgctgaataggattggttctggtctgcctgcagggccgccaaatcataaaaaatttatttgctttgtgagcggataacaattataatagAATTCGAGCTCGGTACCCGGGgatcctctagaaataattttgtttaactttaagaaggagatatacatatggtgagcaagggcgaggagctgttcaccggggtggtgcccatcctggtcgagctggacggcgacgtaaacggccacaagttcagcgtgtccggcgagggcgagggcgatgccacctacggcaagctgaccctgaagttcatctgcaccaccggcaagctgcccgtgccctggcccaccctcgtgaccaccctgacctacggcgtgcagtgcttcagccgctaccccgaccacatgaagcagcacgacttcttcaagtccgccatgcccgaaggctacgtccaggagcgcaccatcttcttcaaggacgacggcaactacaagacccgcgccgaggtgaagttcgagggcgacaccctggtgaaccgcatcgagctgaagggcatcgacttcaaggaggacggca

**>pKESaPar**

ggccgcaaatcataaaaaatttatttgctttgtgagcggataacaattataataggatcctctagaaataattttgtttaactttaagaaggagatatacatatggtgagcaagggcgaggagctgttcaccggggtggtgcccatcctggtcgagctggacggcgacgtaaacggccacaagttcagcgtgtccggcgagggcgagggcgatgccacctacggcaagctgaccctgaagttcatctgcaccaccggcaagctgcccgtgccctggcccaccctcgtgaccaccctgacctacggcgtgcagtgcttcagccgctaccccgaccacatgaagcagcacgacttcttcaagtccgccatgcccgaaggctacgtccaggagcgcaccatcttcttcaaggacgacggcaactacaagacccgcgccgaggtgaagttcgagggcgacaccctggtgaaccgcatcgagctgaagggcatcgacttcaaggaggacggcaacatcctggggcacaagctggagtacaactacaacagccacaacgtctatatcatggccgacaagcagaagaacggcatcaaggtgaacttcaagatccgccacaacatcgaggacggcagcgtgcagctcgccgaccactaccagcagaacacccccatcggcgacggccccgtgctgctgcccgacaaccactacctgagcacccagtccgccctgagcaaagaccccaacgagaagcgcgatcacatggtcctgctggagttcgtgaccgccgccgggatcactctcggcatggacgagctgtacaagtaaagcttggctgttttggcggatgagagaagattttcagcctgatacagattaaatcagaacgcagaagcggtctgataaaacagaatttgcctggcggcagtagcgcggtggtcccacctgaccccatgccgaactcagaagtgaaacgccgtagcgccgatggtagtgtggggtctccccatgcgagagtagggaactgccaggcatcaaataaaacgaaaggctcagtcgaaagactgggcctttcgttttatctgttgtttgtcggtgaacgctctcctgagtaggacaaatccgccgggagcggatttgaacgttgcgaagcaacggcccggagggtggcgggcaggacgcccgccataaactgccaggcatcaaattaagcagaaggccatcctgacggatggcctttttgcgtttctacaaactcttttgtttatttttctaaatacattcaaatatgtatccgctcatgaacaataaaactgtctgcttacataaacagtaatacaaggggtgttatgagccatattcaacgggaaacgtcttgctctaggccgcgattaaattccaacatggatgctgatttatatgggtataaatgggctcgcgataatgtcgggcaatcaggtgcgacaatctatcgattgtatgggaagcccgatgcgccagagttgtttctgaaacatggcaaaggtagcgttgccaatgatgttacagatgagatggtcagactaaactggctgacggaatttatgcctcttccgaccatcaagcattttatccgtactcctgatgatgcatggttactcaccactgcgatccccgggaaaacagcattccaggtattagaagaatatcctgattcaggtgaaaatattgttgatgcgctggcagtgttcctgcgccggttgcattcgattcctgtttgtaattgtccttttaacagcgatcgcgtatttcgtctcgctcaggcgcaatcacgaatgaataacggtttggttgatgcgagtgattttgatgacgagcgtaatggctggcctgttgaacaagtctggaaagaaatgcataaacttttgccattctcaccggattcagtcgtcactcatggtgatttctcacttgataaccttatttttgacgaggggaaattaataggttgtattgatgttggacgagtcggaatcgcagaccgataccaggatcttgccatcctatggaactgcctcggtgagttttctccttcattacagaaacggctttttcaaaaatatggtattgataatcctgatatgaataaattgcagtttcatttgatgctcgatgagtttttctaagaattaattcatgaccaaaatcccttaacgtgagttttcgttccactgagcgtcagacactagtgctactttccgaacgactcctgcgttgatcggaaatccagaagcccgagaggttgccgcctttcgggctttttctttttcaaaaaaaaaaatttataaaacgatctgttgcggccgccgggttgtgggcaaaggcgctcgacggtgggcaaccgcttgcggttgtccacgggcggagccggtgcgcgtagcgcattgtccacaagccaagggcgaccaataattgatatatatattcataattgaaaagctaattgaacatactacttgctgtaactacttgccggagcgaggggtgtttgcaagctgttgatctgaaagggctattagcgttctcacgtgcctttttgattagcgatttcacgtgaccttattagcgatttcacgtactccgattagcgatttcacgtaccctgattagcgatttcacgtggatagtttttggagcgggccggaaagccccgtgaatcaaggctttgcggggcattagcggtttcacgtggataactaccctctatccacaggcttccggggataaaaaagcccgctcgacggcgggctgttggatgggaaggcttgaccaagccaagcgtagcgttggcctggtcaagtcggaggggggccgatgcgagcgcccttgccgggtgcgcgggtgacatgcaggcgtgtggatttgatgcgcaggcattcgccgtcatcttcgatgcagtcgcttgcctcgggatagacaatcaacacttcgcgtaggcgctttttgaagttgtatttgaagctggcgagtgctgcccgctctgcccgctctcgggccttatcgtccagttcgggcgagttgcgtgcgcggctgccataggatgagccgaattgcgcttgcagggcgacccaagggatttgcacgaaggggcggcccttggcccgcaacaggaacacgcgataggtcagccacgtgtaaatgtccatcgcaagcggagactgccgcaaggcatgcaggtagtcgattcggataggaaccggtgagcgggtgacttcctcgaagaaatcgcctgtgagggtgagggtgctatcccatagcgcccgatcttctggccgcttgggattccagaatagaaaagcgcgcttggcaatgacgacgttctcaatgccgaagtcattgccttgctcgccggcaagcgaaatcatggatgaaaacaggcgttgcgcctgattgcgaagggtggccgtgtaacggccatcggtgtgcattccgagcctttgtagaaattccgattgcgaccggccaaggttcaacacggggtctttcgttcgcacggcctcggtgcatatccaagcaagcaaggtgcgcggcatagaaccgtagggcaggccgatgctcggcttgcccatgatcgacaaggtgacgatgccattggtgcgctcaaagtagctggtcttggggtcggtgtggggcatggtcgcttgcacaaggcaacgggccatgtagccgactaagccagcttcgcgggcatcctccatttcgagcgcgaggctcgtcttgatgatctcgttgatacgatggccgggggctttgttgttcttaggcatgttgttccctccccggcatggtgatggttggtctagtgtttgtgggtttgatgttccggcgtttgatgaacaggcgcaaggtgtgagggctgacgcctaacaactcggctgcgcgactttgcggcaagccaaggttcacgtatgcctgtacttcatcaatacggctgtccagcttcaaggcgctcgatttgctgcccttgggtcgcccgagcgtcttgccgcgctctctggcgacttgtagcgcctcggtggtacgtgcctgaatgaaatgccgctcgatctgtgcagccaagccaagcacggttgccatgatgtcgctttgtaggctgccgtccatgatgatcttctgtttggtcacatggacgattaggccgcgctcgctcgccgctttgagaatttccaaggcggcgagggcggaaccggcaatgcgcgtaatctccggcgtcagtagcacgtcgccacgctcggccttttcgatgattgctccgagcttgcgcttgcgccagtcctttgctctgctggcaatttcttcctcgatctgtagcggcgcgaagcctttggcgttcgcgtattcgagcaaaccgtatttttggttttccgggtcttggccgtcacgcgaaacccggagataggcatagtattttggcatttgcagggaaaacgtcagattcggttaaacatgcctcattctagcgcagattaaataggaattaaataccctgtagcggtatagataaaacgttggtttgttctgccctatgagcgtacaaaaaaggccgggtgagtggcccggccttcgtttaggtgctgaataggattggttctggtctgcctgcaggcgaaaaggtgagaaaagccgggcactgcccggctttatttttgctgctgcgcgttccaggccgcccacactcgtttgacctggctcgggctgcatccgaccagcttggccgtcttggcaatgctcgatccgccggagcgaagcgtgatgatgcggtcgtgcatgccggcgtcacgtttgcggccggtgtagcggccggcggccttcgccaactggacaccctgacgttgacgctcgcgccgatcctcgtagtcgtcgcgggccatctgcaaggcgagcttcaaaagcatgtcctggacggattccagaacgattttcgccactccgttcgcctcggcggccagctccgacaggtccaccacgccaggcacggccagcttggcccctttggcccggatcgacgcaaccaggcgctcggcctcggccaacggcaagcggctgatgcggtcgatcttctccgcaacgacgacttcaccaggttgcaggtccgcgatcatgcgcagcagctcgggccggtcggcgcgtgcgccggacgccttctcgcggtagatgccggcgacgtagtacccggcggcccgcgtggccgctacaaggctctcctggcgttcaagattctgctcgtccgtactggcgcgcaggtagatgcgggcgaccttcaaccttcgtccctccggttgttgctctcgcgtcgccatttccacggctcgacggcgtgcggatcggaccagaggccgacgcgcttgcctcgcgcctcctgttcgagccgcagcatttcagggtcggccgcgcggccgtggaagcgataggcccacgccatgccctggtgaaccatcgcggcgttgacgttgcgcggctgcggcggccggctggccagctccatgttgacccacacggtgcccagcgtgcggccgtaacggtcggtgtccttctcgtcgaccaggacgtgccggcggaacaccatgccggccagcgcctggcgcgcacgttcgccgaaggcttgccgcttttccggcgcgtcaatgtccaccaggcgcacgcgcaccggctgcttgtctaccagcacgtcgatggtgtcgccgtcgatgatgcgcacgacctcgccgcgcagctcggcccatgccggcgaggcaacgaccaggacggccagcgcggcagcggcgcgcagcatggcgtagcttcggcgcttcatgcgtggccccattgctgatgatcggggtacgccaggtgcagcactgcatcgaaattggccttgcagtagccgtccagcgccacccgcgagccgaacgccggcgaaaggtactcgaccaggccgggccggtcgcggacctcgcgccccaggacgtggatgcgccggccgcgtgtgccgtcgggtccaggcacgaaggccagcgcctcgatgttgaagtcgatggatagaagttgtcggtagtgcttggccgccctcatcgcgtcccccttggtcaaattgggtatacccatttgggcctagtctagccggcatggcgcattacagcaatacgcaatttaaatgcgcctagcgcattttcccgaccttaatgcgcctcgcgctgtagcctcacgcccacacatgtgctaatgtggttacgtgtattttatggaggttatccaatgagccgcctgacaatcgacatgacggaccagcagcaccagagcctgaaagccctggccgccttgcagggcaagaccattaagcaatacgccctcgaacgtctgttccccggtgacgctgatgccgatcaggcatggcaggaactgaaaaccatgctggggaaccgcatcaacgatgggcttgccggcaaggtgtccaccaagagcgtcggcgaaattcttgatgaagaactcagcggggatcgcgcttgacggcctacatcctcacggctgaggccgaagccgatctacgcggcatcatccgctacacgcgccgggagtggggcgcggcgcaggtgcgccgctatatcgctaagctggaacagggcatagccaggcttgccgccggcgaaggcccgtttaaggacatgagcgaactctttcccgcgctgcggatggcccgctgcgaacaccactacgttttttgcctgccgcgtgcgggcgaacccgcgttggtcgtggcgatcctgcatgagcgcatggacctcatgacgcgacttgccgacaggctcaagggctgaccgcgtcagacgcccgtagcagcccgctacgggctttttcatgccctgcccgc

**>pKERep**

ctgaagggcatcgacttcaaggaggacggcaacatcctggggcacaagctggagtacaactacaacagccacaacgtctatatcatggccgacaagcagaagaacggcatcaaggtgaacttcaagatccgccacaacatcgaggacggcagcgtgcagctcgccgaccactaccagcagaacacccccatcggcgacggccccgtgctgctgcccgacaaccactacctgagcacccagtccgccctgagcaaagaccccaacgagaagcgcgatcacatggtcctgctggagttcgtgaccgccgccgggatcactctcggcatggacgagctgtacaagtaaaagcttggctgttttggcggatgagagaagattttcagcctgatacagattaaatcagaacgcagaagcggtctgataaaacagaatttgcctggcggcagtagcgcggtggtcccacctgaccccatgccgaactcagaagtgaaacgccgtagcgccgatggtagtgtggggtctccccatgcgagagtagggaactgccaggcatcaaataaaacgaaaggctcagtcgaaagactgggcctttcgttttatctgttgtttgtcggtgaacgctctcctgagtaggacaaatccgccgggagcggatttgaacgttgcgaagcaacggcccggagggtggcgggcaggacgcccgccataaactgccaggcatcaaattaagcagaaggccatcctgacggatggcctttttgcgtttctacaaactcttttgtttatttttctaaatacattcaaatatgtatccgctcatgaataataaaactgtctgcttacataaacagtaatacaaggggtgttatgagccatattcaacgggaaacgtcttgctctaggccgcgattaaattccaacatggatgctgatttatatgggtataaatgggctcgcgataatgtcgggcaatcaggtgcgacaatctatcgattgtatgggaagcccgatgcgccagagttgtttctgaaacatggcaaaggtagcgttgccaatgatgttacagatgagatggtcagactaaactggctgacggaatttatgcctcttccgaccatcaagcattttatccgtactcctgatgatgcatggttactcaccactgcgatccccgggaaaacagcattccaggtattagaagaatatcctgattcaggtgaaaatattgttgatgcgctggcagtgttcctgcgccggttgcattcgattcctgtttgtaattgtccttttaacagcgatcgcgtatttcgtctcgctcaggcgcaatcacgaatgaataacggtttggttgatgcgagtgattttgatgacgagcgtaatggctggcctgttgaacaagtctggaaagaaatgcataaacttttgccattctcaccggattcagtcgtcactcatggtgatttctcacttgataaccttatttttgacgaggggaaattaataggttgtattgatgttggacgagtcggaatcgcagaccgataccaggatcttgccatcctatggaactgcctcggtgagttttctccttcattacagaaacggctttttcaaaaatatggtattgataatcctgatatgaataaattgcagtttcatttgatgctcgatgagtttttctaagaattaattcatgaccaaaatcccttaacgtgagttttcgttccactgagcgtcagacactagtgcggccaccggctggctcgcttcgctcggcccgtggacaaccctgctggacaagctgatggacaggctgcgcctgcccacgagcttgaccacagggattgcccaccggctacccagccttcgaccacatacccaccggctccaactgcgcggcctgcggccttgccccatcaatttttttaattttctctggggaaaagcctccggcctgcggcctgcgcgcttcgcttgccggttggacaccaagtggaaggcgggtcaaggctcgcgcagcgaccgcgcagcggcttggccttgacgcgcctggaacgacccaagcctatgcgagtgggggcagtcgaagggcgaagcccgcccgcctgccccccgagcctcacggcggcgagtgcgggggttccaagggggcagcgccaccttgggcaaggccgaaggccgcgcagtcgatcaacaagccccggaggggccactttttgccggagggggagccgcgccgaaggcgtgggggaaccccgcaggggtgcccttctttgggcaccaaagaactagatatagggcgaaatgcgaaagacttaaaaatcaacaacttaaaaaaggggggtacgcaacagctcattgcggcaccccccgcaatagctcattgcgtaggttaaagaaaatctgtaattgactgccacttttacgcaacgcataattgttgtcgcgctgccgaaaagttgcagctgattgcgcatggtgccgcaaccgtgcggcacccctaccgcatggagataagcatggccacgcagtccagagaaatcggcattcaagccaagaacaagcccggtcactgggtgcaaacggaacgcaaagcgcatgaggcgtgggccgggcttattgcgaggaaacccacggcggcaatgctgctgcatcacctcgtggcgcagatgggccaccagaacgccgtggtggtcagccagaagacactttccaagctcatcggacgttctttgcggacggtccaatacgcagtcaaggacttggtggccgagcgctggatctccgtcgtgaagctcaacggccccggcaccgtgtcggcctacgtggtcaatgaccgcgtggcgtggggccagccccgcgaccagttgcgcctgtcggtgttcagtgccgccgtggtggttgatcacgacgaccaggacgaatcgctgttggggcatggcgacctgcgccgcatcccgaccctgtatccgggcgagcagcaactaccgaccggccccggcgaggagccgcccagccagcccggcattccgggcatggaaccagacctgccagccttgaccgaaacggaggaatgggaacggcgcgggcagcagcgcctgccgatgcccgatgagccgtgttttctggacgatggcgagccgttggagccgccgacacgggtcacgctgccgcgccggtagctgcagcccaaatcataaaaaatttatttgctttgtgagcggataacaattataatagAATTCGAGCTCGGTACCCGGGgatcctctagaaataattttgtttaactttaagaaggagatatacatatggtgagcaagggcgaggagctgttcaccggggtggtgcccatcctggtcgagctggacggcgacgtaaacggccacaagttcagcgtgtccggcgagggcgagggcgatgccacctacggcaagctgaccctgaagttcatctgcaccaccggcaagctgcccgtgccctggcccaccctcgtgaccaccctgacctacggcgtgcagtgcttcagccgctaccccgaccacatgaagcagcacgacttcttcaagtccgccatgcccgaaggctacgtccaggagcgcaccatcttcttcaaggacgacggcaactacaagacccgcgccgaggtgaagttcgagggcgacaccctggtgaaccgcatcgag

**>pKERepPar**

ggccgccaaatcataaaaaatttatttgctttgtgagcggataacaattataatagAATTCGAGCTCGGTACCCGGGgatcctctagaaataattttgtttaactttaagaaggagatatacatatggtgagcaagggcgaggagctgttcaccggggtggtgcccatcctggtcgagctggacggcgacgtaaacggccacaagttcagcgtgtccggcgagggcgagggcgatgccacctacggcaagctgaccctgaagttcatctgcaccaccggcaagctgcccgtgccctggcccaccctcgtgaccaccctgacctacggcgtgcagtgcttcagccgctaccccgaccacatgaagcagcacgacttcttcaagtccgccatgcccgaaggctacgtccaggagcgcaccatcttcttcaaggacgacggcaactacaagacccgcgccgaggtgaagttcgagggcgacaccctggtgaaccgcatcgagctgaagggcatcgacttcaaggaggacggcaacatcctggggcacaagctggagtacaactacaacagccacaacgtctatatcatggccgacaagcagaagaacggcatcaaggtgaacttcaagatccgccacaacatcgaggacggcagcgtgcagctcgccgaccactaccagcagaacacccccatcggcgacggccccgtgctgctgcccgacaaccactacctgagcacccagtccgccctgagcaaagaccccaacgagaagcgcgatcacatggtcctgctggagttcgtgaccgccgccgggatcactctcggcatggacgagctgtacaagtaaaagcttggctgttttggcggatgagagaagattttcagcctgatacagattaaatcagaacgcagaagcggtctgataaaacagaatttgcctggcggcagtagcgcggtggtcccacctgaccccatgccgaactcagaagtgaaacgccgtagcgccgatggtagtgtggggtctccccatgcgagagtagggaactgccaggcatcaaataaaacgaaaggctcagtcgaaagactgggcctttcgttttatctgttgtttgtcggtgaacgctctcctgagtaggacaaatccgccgggagcggatttgaacgttgcgaagcaacggcccggagggtggcgggcaggacgcccgccataaactgccaggcatcaaattaagcagaaggccatcctgacggatggcctttttgcgtttctacaaactcttttgtttatttttctaaatacattcaaatatgtatccgctcatgaataataaaactgtctgcttacataaacagtaatacaaggggtgttatgagccatattcaacgggaaacgtcttgctctaggccgcgattaaattccaacatggatgctgatttatatgggtataaatgggctcgcgataatgtcgggcaatcaggtgcgacaatctatcgattgtatgggaagcccgatgcgccagagttgtttctgaaacatggcaaaggtagcgttgccaatgatgttacagatgagatggtcagactaaactggctgacggaatttatgcctcttccgaccatcaagcattttatccgtactcctgatgatgcatggttactcaccactgcgatccccgggaaaacagcattccaggtattagaagaatatcctgattcaggtgaaaatattgttgatgcgctggcagtgttcctgcgccggttgcattcgattcctgtttgtaattgtccttttaacagcgatcgcgtatttcgtctcgctcaggcgcaatcacgaatgaataacggtttggttgatgcgagtgattttgatgacgagcgtaatggctggcctgttgaacaagtctggaaagaaatgcataaacttttgccattctcaccggattcagtcgtcactcatggtgatttctcacttgataaccttatttttgacgaggggaaattaataggttgtattgatgttggacgagtcggaatcgcagaccgataccaggatcttgccatcctatggaactgcctcggtgagttttctccttcattacagaaacggctttttcaaaaatatggtattgataatcctgatatgaataaattgcagtttcatttgatgctcgatgagtttttctaagaattaattcatgaccaaaatcccttaacgtgagttttcgttccactgagcgtcagacactagtgcggccaccggctggctcgcttcgctcggcccgtggacaaccctgctggacaagctgatggacaggctgcgcctgcccacgagcttgaccacagggattgcccaccggctacccagccttcgaccacatacccaccggctccaactgcgcggcctgcggccttgccccatcaatttttttaattttctctggggaaaagcctccggcctgcggcctgcgcgcttcgcttgccggttggacaccaagtggaaggcgggtcaaggctcgcgcagcgaccgcgcagcggcttggccttgacgcgcctggaacgacccaagcctatgcgagtgggggcagtcgaagggcgaagcccgcccgcctgccccccgagcctcacggcggcgagtgcgggggttccaagggggcagcgccaccttgggcaaggccgaaggccgcgcagtcgatcaacaagccccggaggggccactttttgccggagggggagccgcgccgaaggcgtgggggaaccccgcaggggtgcccttctttgggcaccaaagaactagatatagggcgaaatgcgaaagacttaaaaatcaacaacttaaaaaaggggggtacgcaacagctcattgcggcaccccccgcaatagctcattgcgtaggttaaagaaaatctgtaattgactgccacttttacgcaacgcataattgttgtcgcgctgccgaaaagttgcagctgattgcgcatggtgccgcaaccgtgcggcacccctaccgcatggagataagcatggccacgcagtccagagaaatcggcattcaagccaagaacaagcccggtcactgggtgcaaacggaacgcaaagcgcatgaggcgtgggccgggcttattgcgaggaaacccacggcggcaatgctgctgcatcacctcgtggcgcagatgggccaccagaacgccgtggtggtcagccagaagacactttccaagctcatcggacgttctttgcggacggtccaatacgcagtcaaggacttggtggccgagcgctggatctccgtcgtgaagctcaacggccccggcaccgtgtcggcctacgtggtcaatgaccgcgtggcgtggggccagccccgcgaccagttgcgcctgtcggtgttcagtgccgccgtggtggttgatcacgacgaccaggacgaatcgctgttggggcatggcgacctgcgccgcatcccgaccctgtatccgggcgagcagcaactaccgaccggccccggcgaggagccgcccagccagcccggcattccgggcatggaaccagacctgccagccttgaccgaaacggaggaatgggaacggcgcgggcagcagcgcctgccgatgcccgatgagccgtgttttctggacgatggcgagccgttggagccgccgacacgggtcacgctgccgcgccggtagctgcaggcgaaaaggtgagaaaagccgggcactgcccggctttatttttgctgctgcgcgttccaggccgcccacactcgtttgacctggctcgggctgcatccgaccagcttggccgtcttggcaatgctcgatccgccggagcgaagcgtgatgatgcggtcgtgcatgccggcgtcacgtttgcggccggtgtagcggccggcggccttcgccaactggacaccctgacgttgacgctcgcgccgatcctcgtagtcgtcgcgggccatctgcaaggcgagcttcaaaagcatgtcctggacggattccagaacgattttcgccactccgttcgcctcggcggccagctccgacaggtccaccacgccaggcacggccagcttggcccctttggcccggatcgacgcaaccaggcgctcggcctcggccaacggcaagcggctgatgcggtcgatcttctccgcaacgacgacttcaccaggttgcaggtccgcgatcatgcgcagcagctcgggccggtcggcgcgtgcgccggacgccttctcgcggtagatgccggcgacgtagtacccggcggcccgcgtggccgctacaaggctctcctggcgttcaagattctgctcgtccgtactggcgcgcaggtagatgcgggcgaccttcaaccttcgtccctccggttgttgctctcgcgtcgccatttccacggctcgacggcgtgcggatcggaccagaggccgacgcgcttgcctcgcgcctcctgttcgagccgcagcatttcagggtcggccgcgcggccgtggaagcgataggcccacgccatgccctggtgaaccatcgcggcgttgacgttgcgcggctgcggcggccggctggccagctccatgttgacccacacggtgcccagcgtgcggccgtaacggtcggtgtccttctcgtcgaccaggacgtgccggcggaacaccatgccggccagcgcctggcgcgcacgttcgccgaaggcttgccgcttttccggcgcgtcaatgtccaccaggcgcacgcgcaccggctgcttgtctaccagcacgtcgatggtgtcgccgtcgatgatgcgcacgacctcgccgcgcagctcggcccatgccggcgaggcaacgaccaggacggccagcgcggcagcggcgcgcagcatggcgtagcttcggcgcttcatgcgtggccccattgctgatgatcggggtacgccaggtgcagcactgcatcgaaattggccttgcagtagccgtccagcgccacccgcgagccgaacgccggcgaaaggtactcgaccaggccgggccggtcgcggacctcgcgccccaggacgtggatgcgccggccgcgtgtgccgtcgggtccaggcacgaaggccagcgcctcgatgttgaagtcgatggatagaagttgtcggtagtgcttggccgccctcatcgcgtcccccttggtcaaattgggtatacccatttgggcctagtctagccggcatggcgcattacagcaatacgcaatttaaatgcgcctagcgcattttcccgaccttaatgcgcctcgcgctgtagcctcacgcccacacatgtgctaatgtggttacgtgtattttatggaggttatccaatgagccgcctgacaatcgacatgacggaccagcagcaccagagcctgaaagccctggccgccttgcagggcaagaccattaagcaatacgccctcgaacgtctgttccccggtgacgctgatgccgatcaggcatggcaggaactgaaaaccatgctggggaaccgcatcaacgatgggcttgccggcaaggtgtccaccaagagcgtcggcgaaattcttgatgaagaactcagcggggatcgcgcttgacggcctacatcctcacggctgaggccgaagccgatctacgcggcatcatccgctacacgcgccgggagtggggcgcggcgcaggtgcgccgctatatcgctaagctggaacagggcatagccaggcttgccgccggcgaaggcccgtttaaggacatgagcgaactctttcccgcgctgcggatggcccgctgcgaacaccactacgttttttgcctgccgcgtgcgggcgaacccgcgttggtcgtggcgatcctgcatgagcgcatggacctcatgacgcgacttgccgacaggctcaagggctgaccgcgtcagacgcccgtagcagcccgctacgggctttttcatgccctgcccgc

**References**

1. Gruber S, Hagen J, Schwab H, Koefinger P. Versatile and stable vectors for efficient gene expression in *Ralstonia eutropha* H16. J Biotechnol. 2014 Sep 30;186:74–82.

2. Lambauer V, Kratzer R. Lab-scale cultivation of *Cupriavidus necator* on explosive gas mixtures: Carbon dioxide fixation into polyhydroxybutyrate. Bioengineering. 2022 May 1;9(5):204.
